# Inhibition of TGM2 enhances cisplatin sensitivity in MSH2-deficient bladder cancer

**DOI:** 10.1038/s41420-026-03182-z

**Published:** 2026-05-28

**Authors:** Wenjie Wei, Xingyuan Xiao, Chao Ren, Hui Zhang, Jiayin Sun, Miao Wang, Liang Wang, Hebing Chen, Guosong Jiang, Chao Huang

**Affiliations:** 1 Department of Urology, Union Hospital, Tongji Medical College, Huazhong University of Science and Technology, Wuhan, China; 2 Department of Urology, The Affiliated Hospital of Qingdao University, Qingdao, China; 3 Department of Urology, Zhongnan Hospital of Wuhan University, Wuhan, China; 4 Academy of Military Medical Sciences, Beijing, China, Beijing, China

**Keywords:** Cancer therapy, Cell death

## Abstract

MSH2 (mutS homolog 2) gene, a key component of the DNA mismatch repair system, garners significant attention for its influence on cancer progression and prognosis. Loss of MSH2 protein decreases the chemosensitivity of bladder cancer (BCa) to cisplatin (CDDP). However, precision therapeutic strategy based on MSH2 deficiency is not available clinically. Herein, we reported that knockout of MSH2 reduced the sensitivity of BCa to CDDP. Importantly, GK921, a TGM2-specific inhibitor, increased tumor cell killing by CDDP in MSH2-deficient BCa. GK921 also promoted the chemotherapeutic sensitivity of Msh2-knockout mice to CDDP. In addition, Hi-C analysis indicated that MSH2 deficiency rewired chromatin accessibility of the TGM2 promoter region, leading to recruit more transcription factors. Accordingly, we found that the enrichment levels of transcription factor AP-1 in TGM2 promoter region were increased in MSH2-knockout BCa cells, thereby promoting the expression of TGM2 transcriptionally. This study uncovers that CDDP effectiveness depends on TGM2 levels in MSH2-deficient BCa and that the combination of CDDP with TGM2 inhibition may represent a promising therapeutic strategy for MSH2-deficient BCa patients.

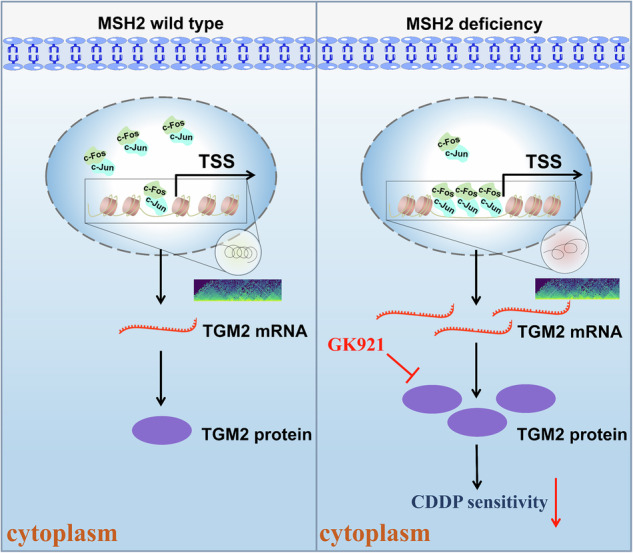

## Introduction

Bladder cancer (BCa) is one of the most common malignancies of the genitourinary system worldwide, and its incidence and mortality have increased in recent years [1]. Clinically, BCa exhibits rapid progression and a high recurrence rate, and 25–30% of BCa cases are considered as muscle-invasive or metastatic disease at the time of diagnosis [2]. Despite the advent of immunotherapy recently, cisplatin (CDDP)-based chemotherapy remains the standard of care for first-line treatment in both neoadjuvant and adjuvant chemotherapy for advanced BCa. However, both of the intrinsic and the acquired CDDP resistance is frequently encountered in clinical practice and has been linked to therapeutic failure and development of distant metastasis [3]. Therefore, uncovering the underlying mechanisms of the cisplatin resistance is urgently needed, which may represent a major step forward in optimizing patients’ outcomes.

Activation status of some DNA damage repair (DDR) genes is robustly correlated with the efficacy of CDDP chemotherapy in BCa. Clinical researches indicate that inactivating mutations in ATM, RB1, FANCC, and ERCC2 have improved response to CDDP chemotherapy in BCa patients [4, 5]. Inactivating mutations in MSH2, a core gene involving in DNA mismatch repair (MMR), could lead to Lynch syndrome, and it is also one of the main genetic-related urothelial cancer pathogenic factors [6–8]. According to genome-wide CRISPR screen, MSH2 is the most significantly enriched candidate gene that promotes resistance of BCa cells to CDDP [9]. Moreover, our previous study demonstrates that the BCa tissues of patients with low MSH2 expression are significantly resistant to CDDP in patient-derived xenografts (PDX) model [10]. Therefore, it is of unparalleled importance to search for the potential resistance mechanisms for BCa with MSH2 protein loss.

Precision medicine, also named personalized medicine, is an approach to identify individualized therapeutic options and to assist guide clinical decision making [11]. The integration of genome sequencing, proteomics, bioinformatics, and big data analysis enables the exploration of disease etiology, precise classification, and molecular-level therapeutic targets, ultimately advancing the realization of personalized medicine and enhancing disease prevention and treatment outcomes [12–14]. An increasing number of molecularly guided treatment options (MGTOs) have gained regulatory approval on the basis of genomic biomarkers across diverse tumor types [14, 15]. The use of PARP inhibitors in BRCA-mutant breast cancer is a classic clinical example of utilizing precision medicine methods to kill tumors [16]. In addition, accumulating evidences have shown that precision treatment strategies based on the dysfunctional MMR genes show promising potential for clinical application. For example, inhibition of particular DNA polymerases can lead to tumor cell death for cancers deficient in the MSH2 or MLH1 [17]. Repression of PRKDC robustly induces apoptosis and displays remarkable efficacy against MSH3-mutant tumors [18]. However, precision oncology approaches to treat bladder tumors with certain MMR components deficiency remain to be revealed.

Herein, we confirmed that loss of MSH2 decreases the chemosensitivity of BCa cells to CDDP. TGM2 is upregulated in MSH2-deficient BCa upon CDDP treatment. Inhibition of TGM2 by knockdown of TGM2 or treatment with GK921 improves the toxicity of CDDP in MSH2-deficient BCa cells. Mechanistically, knockout of MSH2 alters 3D genomic features and increases chromatin accessibility at the TGM2 promoter. This results in elevated binding of AP-1 transcription factors to the TGM2 promoter, promoting its transcriptional activation. Importantly, GK921 could increase tumor cell killing by CDDP in multiple preclinical models of MSH2-deficient BCa. Collectively, this study delineates a novel precision medicine approach for MSH2-deficient BCa.

## Results

### Knockout of MSH2 decreases the sensitivity of BCa to CCDP

**Fig. 1:**
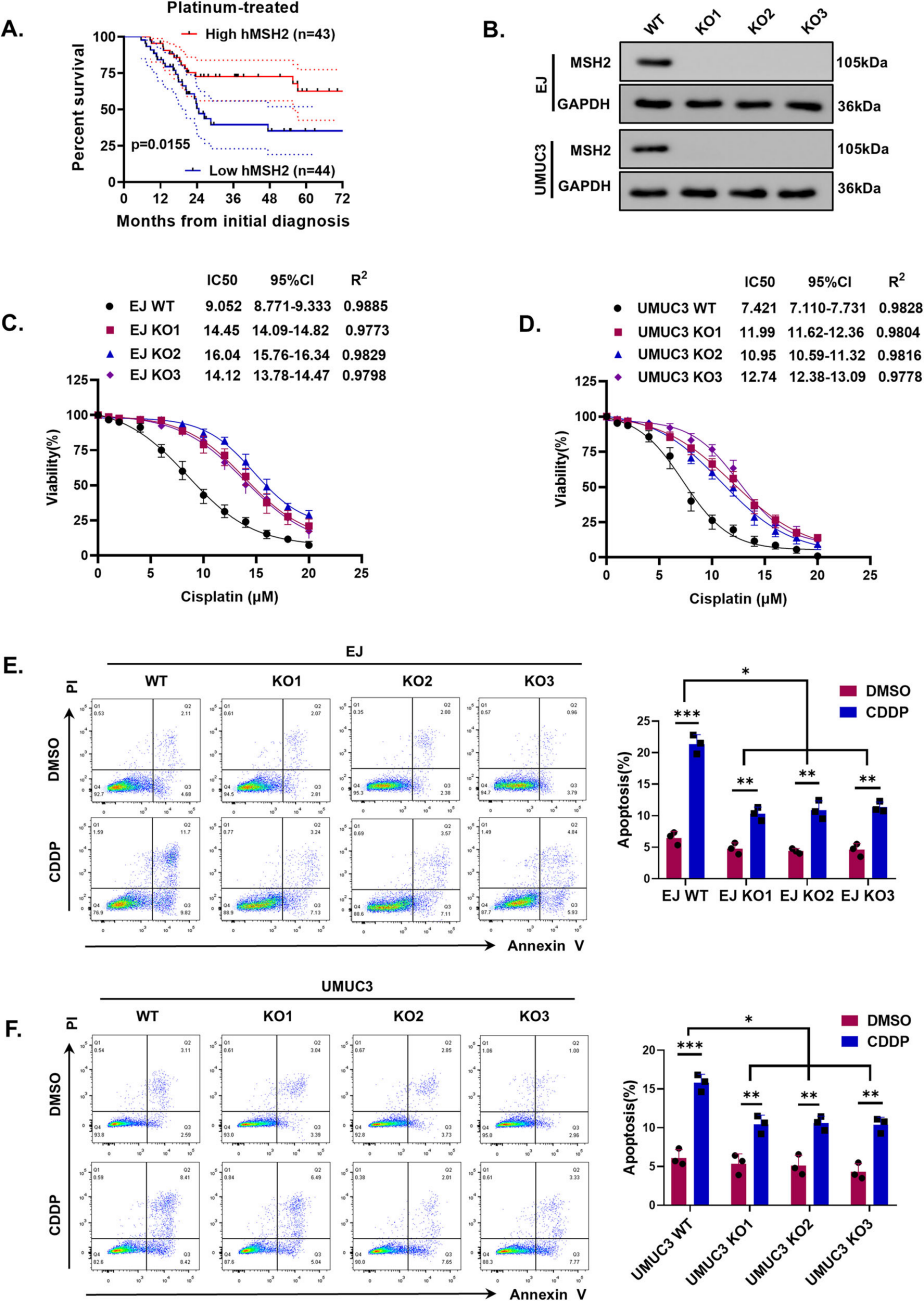
Knockout of MSH2 alleviates the sensitivity of BCa cells to CDDP. **A** Overall survival was plotted for bladder cancer patients with a platinum-based treatment. The 87 patients were divided into 2 equal groups according to the MSH2 protein z-scores from TCGA database. *P*-value was calculated using the log-rank test. **B** Western blot analysis showed the MSH2 levels in MSH2-WT and MSH2-KO bladder cancer cells. **C** Determination of IC50 values for CDDP treatment 24 h in MSH2-WT and MSH2-KO EJ cells (*n* = 3). **D** Determination of IC50 values for CDDP treatment 24 h in MSH2-WT and MSH2-KO UMUC3 cells (*n* = 3). **E** Annexin-V plus PI staining analysis showed the apoptosis rate in MSH2-WT and MSH2-KO EJ cells administered with CDDP (5 μM) for 24 h (*n* = 3). **F** Annexin-V plus PI staining analysis exhibited the apoptosis rate in MSH2-WT and MSH2-KO UMUC3 cells administered with CDDP (5 μM) for 24 h (*n* = 3). Error bars represent standard deviations of the mean obtained from three independent experiments. *P < 0.05, **P < 0.01, ***P < 0.001 by one-way ANOVA in (**E**, **F**).

When MMR is deficient, despite platinum-based chemotherapy can cause DNA damage, cells continue to proliferate and thus have resistance [19, 20]. The MSH2 gene is considered to be one of the most important components of the MMR system. According to the reverse-phase protein arrays of TCGA database, we divided BCa patients into two equal groups based on their MSH2 protein z-scores, and found that patients who received platinum-based therapy with low expression of MSH2 protein had a poor OS (Fig. 1A). To further explore the CDDP sensitivity of BCa involving MSH2 protein loss, we used CRISPR/cas9 gene-editing technology to establish MSH2-knockout EJ and UMUC3 cell lines. The knockout efficacy was verified by western blot (Fig. 1B). The IC50 value of CDDP was increased when MSH2 was knocked out in BCa cells (Fig. 1C, D). Consistently, knockout of MSH2 decreased the CDDP-induced apoptosis in BCa cells (Fig. 1E, F). Collectively, our findings demonstrated that loss of MSH2 decreased the chemosensitivity of BCa to CDDP.

### TGM2 is highly expressed in MSH2-deficient BCa cells

**Fig. 2:**
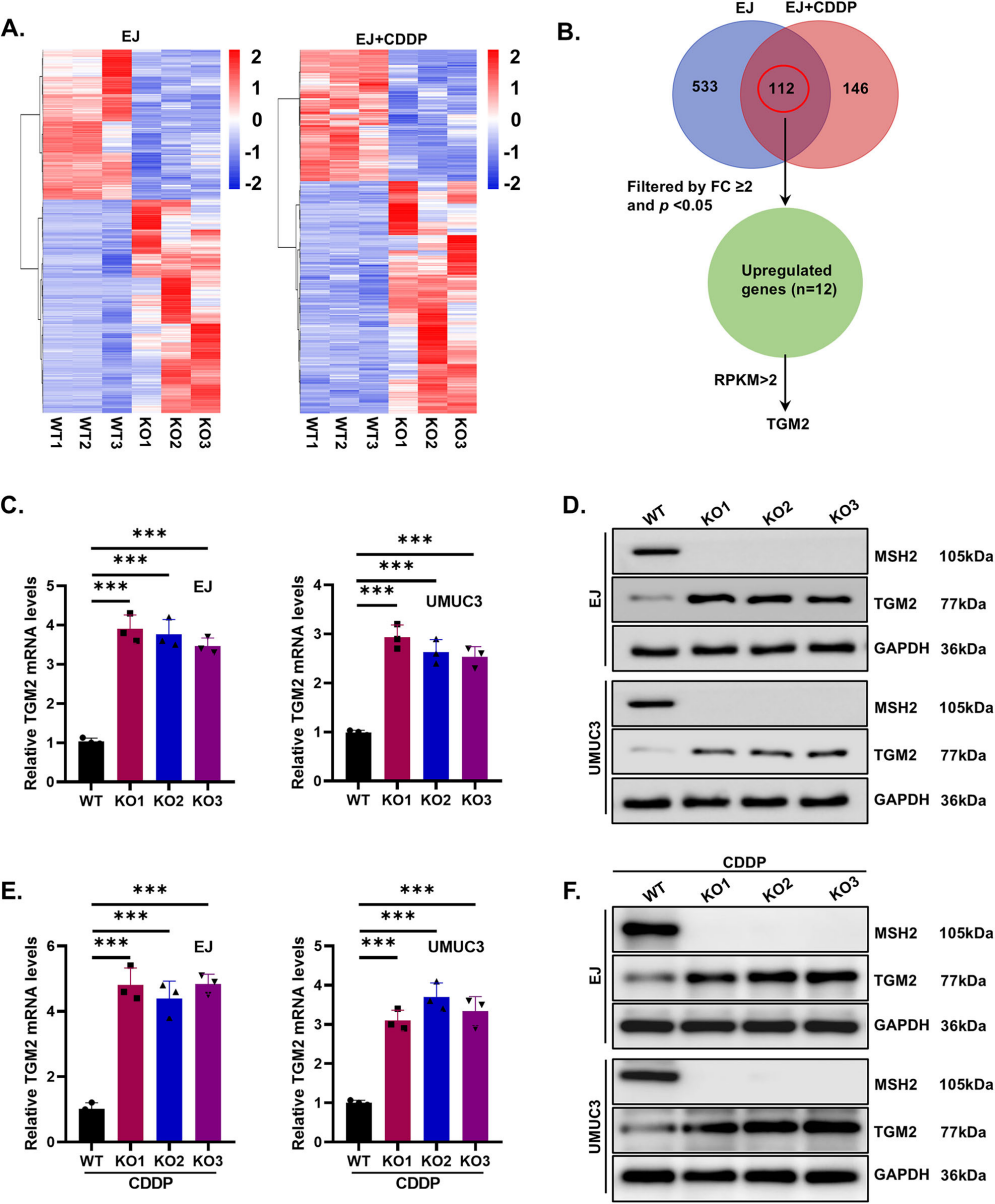
TGM2 is upregulated in MSH2-deficient BCa cells. **A** Heatmap revealed the differentially expressed mRNAs in MSH2-WT and MSH2-KO EJ cells treated with or without CDDP (5 μM). **B** Flowchart depicted that TGM2 was identified as the most significantly upregulated mRNA in our RNA-seq results. **C** The levels of TGM2 mRNA were analyzed by qRT-PCR in MSH2-WT and MSH2-KO bladder cancer cells (*n* = 3). **D** Cell lysates from MSH2-WT and MSH2-KO bladder cancer cells were analyzed by western blotting using TGM2 and GAPDH specific antibodies. **E** The levels of TGM2 mRNA were analyzed by qRT-PCR in MSH2-WT and MSH2-KO bladder cancer cells treated with CDDP (5 μM) for 24 h (*n* = 3). **F** Cell lysates from MSH2-WT and MSH2-KO bladder cancer cells treated with CDDP (5 μM) for 24 h were analyzed by western blotting using TGM2 and GAPDH specific antibodies. Error bars represent standard deviations of the mean obtained from three independent experiments. ***P < 0.001 by one-way ANOVA in (**C**, **E**).

To further explore the potential mechanisms of MSH2 in BCa chemosensitivity, transcriptome analysis of three paired MSH2-deficient cells and matched wild-type cells, administrating with or without CDDP, were performed to explore the changes in gene expression after MSH2 knockout (Fig. 2A). Overlapping with these differentially upregulated mRNAs, TGM2 was the most significant gene filtered by log_2_(FC) ≥ 1, *P* < 0.05 and RPKM > 2 (Fig. 2B). Accordingly, we confirmed that the expression levels of TGM2 mRNA and protein were increased in MSH2-deficient cells (Fig. 2C, D). Furthermore, TGM2 was elevated in MSH2-deficient cells upon CDDP treatment (Fig. 2E, F). Taken together, these results suggested that TGM2 may be a downstream regulatory core gene of MSH2 deficiency in BCa.

According to the TCGA bladder cancer (BLCA) cohort, we found that the expression of TGM2 protein was negatively correlated with MSH2 expression (Fig. S1A). Notably, a similar inverse relationship was observed in live hepatocellular carcinoma (LIHC) and colorectal Adenocarcinoma (COADREAD) cohorts (Fig. S1B, C). However, there is no significant correlation between the expression of TGM2 and MSH2 in lung adenocarcinoma (LUAD), kidney renal clear cell carcinoma (KIRC) and pancreatic adenocarcinoma (PAAD) cohorts (Fig. S1D–F). Taken together, these results suggested that the differential expression of TGM2 in MSH2-deficient malignancies was tissue-specific.

### TGM2 inhibition promotes chemosensitivity of MSH2-deficient BCa to CDDP

**Fig. 3:**
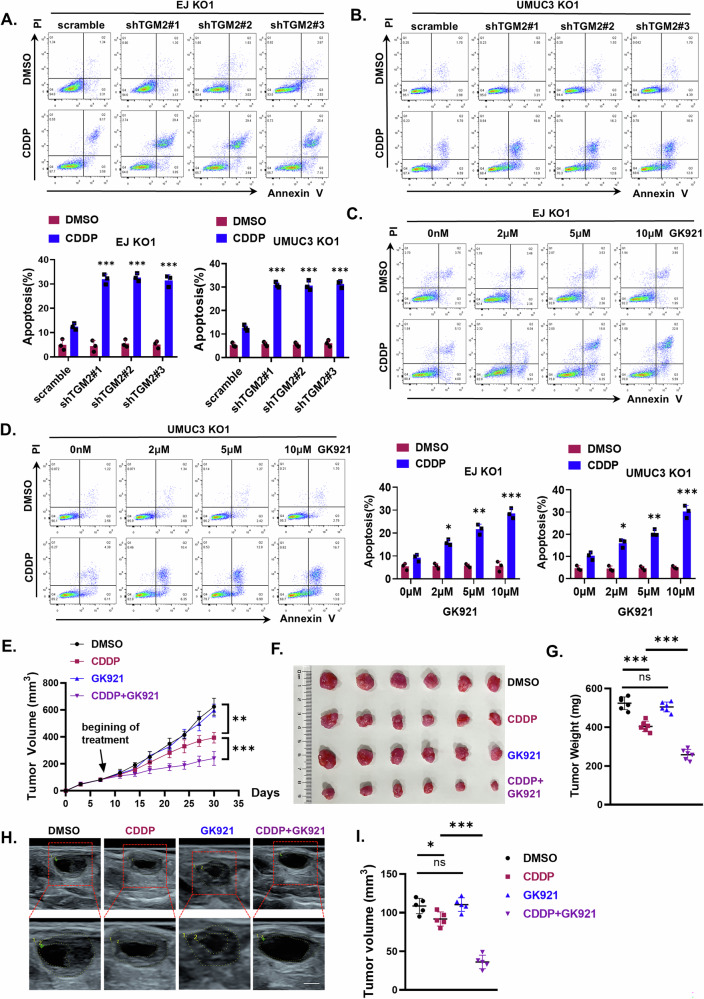
TGM2 inhibition promotes chemosensitivity of MSH2-deficient BCa to CDDP. **A**, **B** MSH2-KO bladder cancer cells were stably transfected with scramble, shTGM2#1, shTGM2#2 or shTGM2#3. Annexin-V plus PI staining analysis depicted the cell apoptosis rate in MSH2-KO bladder cancer cells treated with DMSO or CDDP (5 μM) for 24 h (*n* = 3). **C**, **D** MSH2-KO bladder cancer cells were treated with a series of concentrations of GK921 (0, 2, 5, 10 µM). Annexin-V plus PI staining analysis showed the cell apoptosis rate in MSH2-KO bladder cancer cells treated with DMSO or CDDP (5 μM) for 24 h (*n* = 3). **E** Response of MSH2-KO1 EJ xenografts to treatment with DMSO, CDDP or GK921. Tumor growth curves for each treatment group (*n* = 6). **F** Xenograft tumors of sacrificed mice at the end of the experiment were shown (*n* = 6). **G** Tumor weights for each treatment group (*n* = 6). **H**, **I** The ultrasound images of orthotopic xenograft bladder tumor model were established by MSH2-KO1 EJ cells, along with DMSO, CDDP or GK921 treatment. The low echo area with irregular surface between two lines represented the tumor and the echo free area inside the yellow line 2 was the urine in urinary bladder. Yellow line 1, the wall of urinary bladder; Yellow line 2, the convex surface of tumor toward the bladder lumen (*n* = 5). Scale bar: 1 mm. Error bars represent standard deviations of the mean obtained from three independent experiments. *P < 0.05, **P < 0.01, ***P < 0.001 by one-way ANOVA in (**A**–**E**, **G**, **I**). ns not significant.

Previous studies have supported that genes expression may be the result of functional compensation or “buffering” [21, 22]. Therefore, we speculated that the upregulation of TGM2 expression in MSH2-deficient cells reflected such a “buffering” process. To further validate our hypothesis, we designed and established the short hairpin RNAs (shRNAs) targeting the CDS region of TGM2 (Fig. S2A, B). Intriguingly, short hairpin RNA-induced TGM2 gene silencing sensitized MSH2-deficient BCa cells to CDDP (Fig. 3A, B). In addition, GK921, a TGM2-specific inhibitor [23], promoted CDDP-induced apoptosis in MSH2-deficient BCa cells in a concentration-dependent manner (Fig. 3C, D). TGM2 is known to be involved in processes such as tumor cell invasion and migration [24, 25]. Subsequently, we investigated whether its high expression could promote the metastasis of MSH2-deficient tumors. As shown in Fig. S2C, knockout of MSH2 did not affect the migration of bladder cancer cells. Furthermore, elevated TGM2 levels also did not significantly influence tumor cell migration (Fig. S2D–F). Hence, these results suggested that inhibition of TGM2 could promote the chemosensitivity of MSH2-deficient cells to CDDP.

To further explore whether TGM2 inhibitor is an alternative therapeutic strategy that could improve the effect of CDDP therapy in MSH2-deficient tumors in vivo, MSH2-knockout EJ cells were injected subcutaneously into BALB/c nude mice. The injected cells were allowed to grow for one week to establish tumors. Then the mice were randomly assigned into four groups and were administered with DMSO, CDDP or GK921, and the tumor growth rate was monitored continuously up to 30 days. The results showed that GK921 alone had no effect on tumor growth delaying effect on the xenografts of MSH2-knockout EJ cells. However, combining CDDP with GK921 caused regression of the xenografts transplanted MSH2-deficient EJ cells (Fig. 3E–G).

In addition, given that the subcutaneous model cannot faithfully recapitulate the microenvironment of BCa, we constructed the orthotopic xenograft model of bladder tumor in immunocompromised mice, followed by DMSO, CDDP or GK921 treatment. Subsequent growth of bladder tumors was confirmed and monitored by abdominal ultrasound. Supporting the results obtained in vitro, orthotopic transplantation of MSH2-deficient EJ cells treated with CDDP and GK921 simultaneously displayed smaller tumor size compared with CDDP treatment alone (Fig. 3H, I). In general, our findings demonstrated that loss of TGM2 specifically sensitized MSH2-deficient BCa to CDDP, indicating a precise approach for MSH2-deficient BCa administrated with CDDP.

### TGM2 inhibitor GK921 suppressed the growth of Msh2-null BCa

**Fig. 4:**
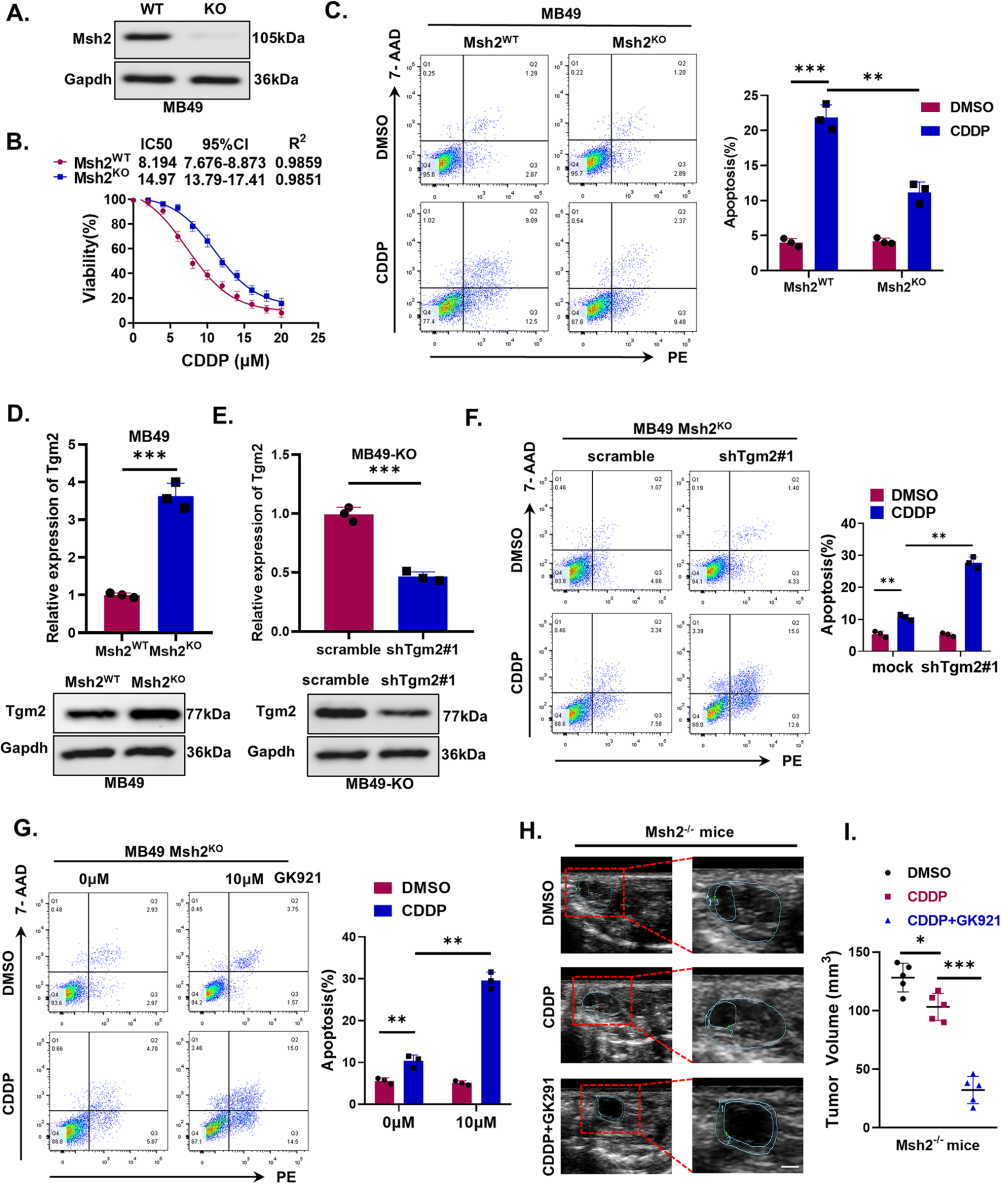
TGM2 inhibitor increases the CDDP toxicity in Msh2-deficient tumor. **A** Western blot analysis showed the Msh2 levels in Msh2-WT and Msh2-KO MB49 cells. **B** Determination of IC50 values of Msh2-WT and Msh2-KO MB49 cells administered with CDDP for 24 h (*n* = 5). **C** Msh2-WT and Msh2-KO MB49 cells were treated with DMSO or CDDP (5 μM) for 24 h, and apoptosis rate was measured by Annexin V-PE/7-AAD staining analysis (*n* = 3). **D** The expression levels of Tgm2 in Msh2-WT and Msh2-KO MB49 cells were detected by qRT-PCR and western blot (*n* = 3). **E** The expression levels of Tgm2 in Msh2-deficient MB49 cells stably transfected with scramble or shTgm2#1 were detected by qRT-PCR and western blot (*n* = 3). **F** Msh2-KO MB49 cells were stably transfected with scramble or shTgm2#1, and were treated with DMSO or CDDP (5 μM) for 24 h. Apoptosis rate was measured by Annexin V-PE plus 7-AAD staining assay (*n* = 3). **G** Msh2-KO MB49 cells were administered with GK921(0, 10 μM). Annexin V-PE plus 7-AAD staining assay showed the apoptosis rate of Msh2-KO MB49 cells treated with DMSO or CDDP (5 μM) for 24 h (*n* = 3). **H**, **I** The ultrasound images of orthotopic bladder tumor model were established by feeding 0.05% BBN to the Msh2^-/-^ mice, along with DMSO, CDDP or GK921 treatment. The low echo area with irregular surface between two lines represented the tumor and the echo free area inside the irregular line of the inner circle was the urine in urinary bladder. Irregular line of the outer circle, the wall of urinary bladder; Irregular line of the inner circle, the convex surface of tumor toward the bladder lumen (*n* = 5). Scale bar: 1 mm. Error bars represent standard deviations of the mean obtained from three independent experiments. *P < 0.05, **P < 0.01, ***P < 0.001 by *t* test in **D**, **E**, one-way ANOVA in (**C**, **F**, **G**, **I**).

Then, we performed CRISPR/cas9 technology to abolish the *Msh2* gene in MB49 cells (Fig. 4A), which is a murine transitional cell line of the BCa. MSH2-deficient MB49 cells exhibited higher IC50 value to CDDP (Fig. 4B). As shown in Fig. 4C, knockout of MSH2 decreased the chemosensitivity of MB49 cells to CDDP. Moreover, loss of MSH2 could also facilitate the expression of TGM2 (Fig. 4D). Then, we constructed MSH2-deficient MB49 cell lines stably transfected with shTGM2 plasmid, and found that silencing of TGM2 markedly increased the apoptosis rate in MSH2-deficient MB49 cells treated with CDDP (Fig. 4E, F). In addition, GK921 also promoted CDDP-induced apoptosis in MSH2-deficient MB49 cells (Fig. 4G).

To further validate these findings, we evaluated the efficacy of GK921 in MSH2-deficient bladder cancer upon CDDP treatment in vivo, which was conducted with the *Msh2*-knockout C57BL/6 J mice. The strategies for the production of *Msh2*-knockout mice and PCR genotyping of offspring were depicted in Fig. S3A–C. The mice were supplied *ad libitum* with ddH_2_O containing 0.05% BBN [26] in opaque bottles for 23 weeks to establish orthotopic tumor models, and were subsequently treated with DMSO, CDDP or GK921. There was a minimal effect in tumor growth upon CDDP alone in Msh2^-/-^ mice, while the near-complete resolution of the orthotopic tumor was noted following the combination therapy of CDDP and GK921 (Fig. 4H, I). Collectively, our results indicated that GK921 increased tumor cell killing by CDDP in MSH2-deficient bladder tumor.

### MSH2 deficiency promotes chemoresistance through AP-1/TGM2 axis

**Fig. 5:**
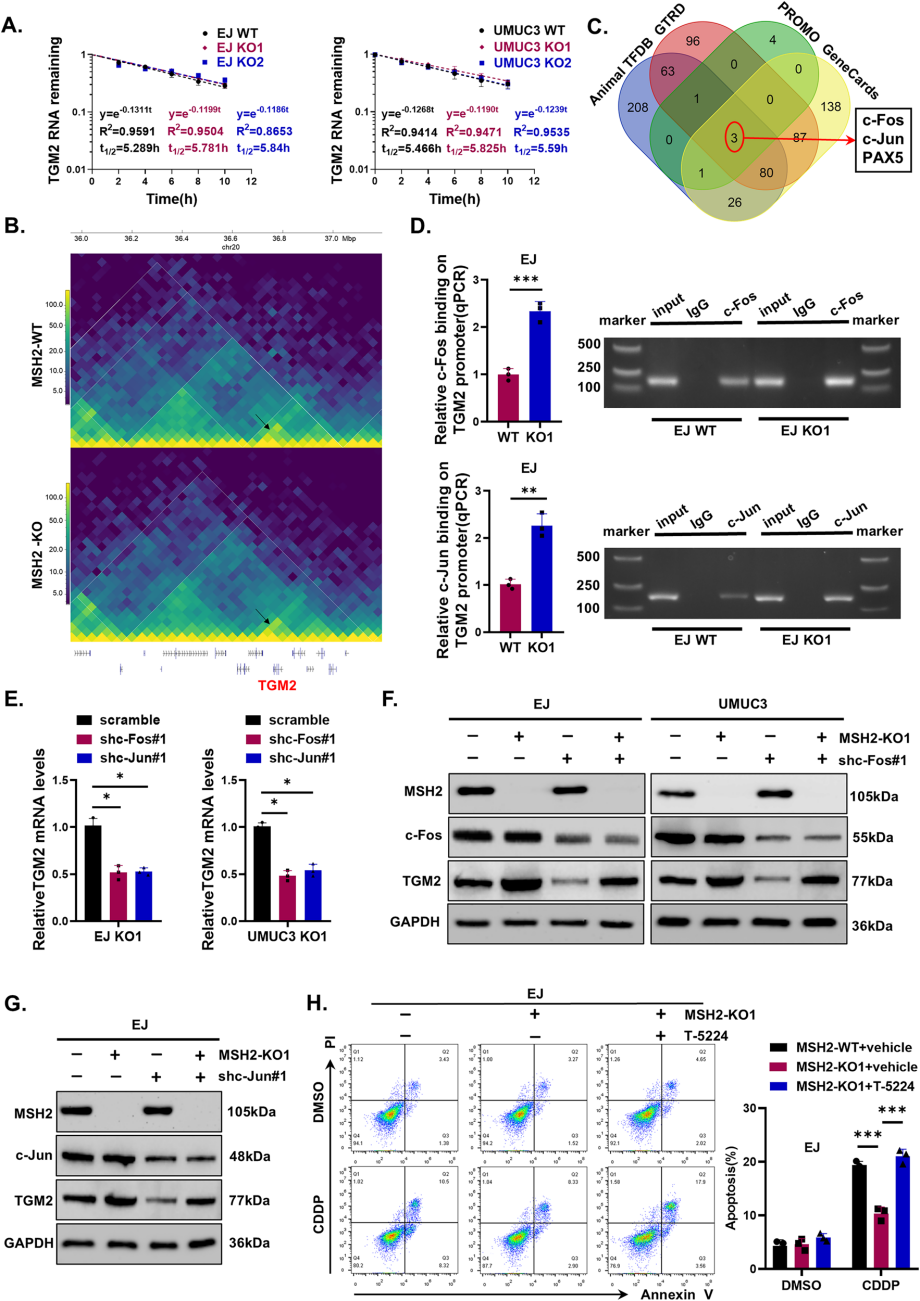
MSH2 deficiency promotes chemoresistance through AP-1/TGM2 axis. **A** Actinomycin D assay showed the stability of TGM2 mRNA in MSH2-WT and MSH2-KO BCa cells (*n* = 3). **B** Hi-C analysis indicated that knockout of MSH2 pioneered the loss of TAD around TGM2. **C** Venn diagram revealed the potential transcription factors of TGM2 promoter predicted by Animal TFDB, GTRD, GeneCards and PROMO databases. **D** ChIP and qPCR assays showed the changes in binding of c-Fos to TGM2 promoter in MSH2-WT and MSH2-KO EJ cells, and the changes in binding of c-Jun to TGM2 promoter in MSH2-WT and MSH2-KO EJ cells (*n* = 3). **E** qRT-PCR assays showed the relative levels of TGM2 mRNA in MSH2-KO BCa cells stably transfected with scramble, shc-Fos#1 or shc-Jun#1 (*n* = 3). **F** Western blot assays showed the TGM2 protein levels in MSH2-WT or MSH2-KO BCa cells, and those transfected with scramble or shc-Fos#1. **G** Western blot assays showed the TGM2 protein levels in MSH2-WT or MSH2-KO EJ cells, and those transfected with scramble or shc-Jun#1. **H** MSH2-WT and MSH-KO1 EJ cells were treated with vehicle or T-5224 at a concentration of 10 µM. Annexin-V plus PI staining analysis showed the cell apoptosis rate in EJ cells treated with DMSO or CDDP (5 μM) for 24 h (*n* = 3). Error bars represent standard deviations of the mean obtained from three independent experiments. *P < 0.05, **P < 0.01, ***P < 0.001 by *t* test in **D**, one-way ANOVA in (**E**, **H**).

We further explore the underlying mechanisms that regulate the increased of TGM2 in MSH2-deficent BCa. mRNA stability assay was performed, and the mRNA decay rates of TGM2 had no significant differences between MSH2-deficient and wild-type BCa cells (Fig. 5A). Therefore, we speculated that the expression change of TGM2 was at the transcriptional level. Chromatin conformation is emerging as an important mechanism to regulate gene expression by altering accessibility of chromatin to transcription factors [27]. Previous study has reported an unexpected role of MSH2 in regulating the expression of cell adhesion-related genes by altering chromatin architecture [28]. Therefore, we conducted Hi-C analysis and found that knockout of MSH2 could result in changes of 3D genomic features in a wide range in chr20:36.0Mbp-38.0 Mbp. The loss of topologically associating domains (TADs) around TGM2 led to the sparser chromatin and higher chromatin accessibility, which made a more frequent recruitment of transcription factors in the promoter of TGM2 (Fig. 5B and Fig. S4A–F). Overlapping analysis of transcription factors of TGM2 promoter derived from Animal TFDB, GTRD, PROMO and GeneCards databases identified three potential trans-acting factors that might regulate the transcription of TGM2 (Fig. 5C), including c-Fos, c-Jun and PAX5. Further validating ChIP-qPCR experiments showed that knockout of MSH2 led to a considerable increase of c-Fos and c-Jun recruitment to TGM2 promoter region (Fig. 5D and Fig. S5A), but not PAX5 (Fig. S5B). The predominant forms of AP-1 family transcription factors in mammalian cells are c-Fos/c-Jun heterodimers, which could bind to the AP-1 site and regulate gene expression [29]. Next, we examined whether inhibition of AP-1 by shRNA could regulate TGM2 expression levels. Accordingly, we found that silencing of c-Fos/c-Jun decreased the mRNA levels of TGM2 in MSH2-deficient BCa cells (Fig. 5E and Fig. S5C). Furthermore, the increasing levels of TGM2 protein in MSH2-deficient BCa cells could be reversed by c-Fos/c-Jun knockdown (Fig. 5F, G and Fig. S5D). T-5224, a small molecule inhibitor of AP-1 [30], downregulated TGM2 in MSH2-deficient bladder cancer (Figure. S5E). Furthermore, as shown in Fig. 5H and Fig. S5F, MSH2 deficiency conferred resistance to CDDP in bladder cancer cells, which could be abrogated by T-5224 treatment. Collectively, these findings demonstrated that loss of MSH2 upregulated TGM2 through recruiting more AP-1 to the its promoter, thereby enhancing chemoresistance to CDDP in bladder cancer cells.

### MSH2 loss potentiates chemoresistance via TGM2-mediated enhancement of DNA damage repair

**Fig. 6:**
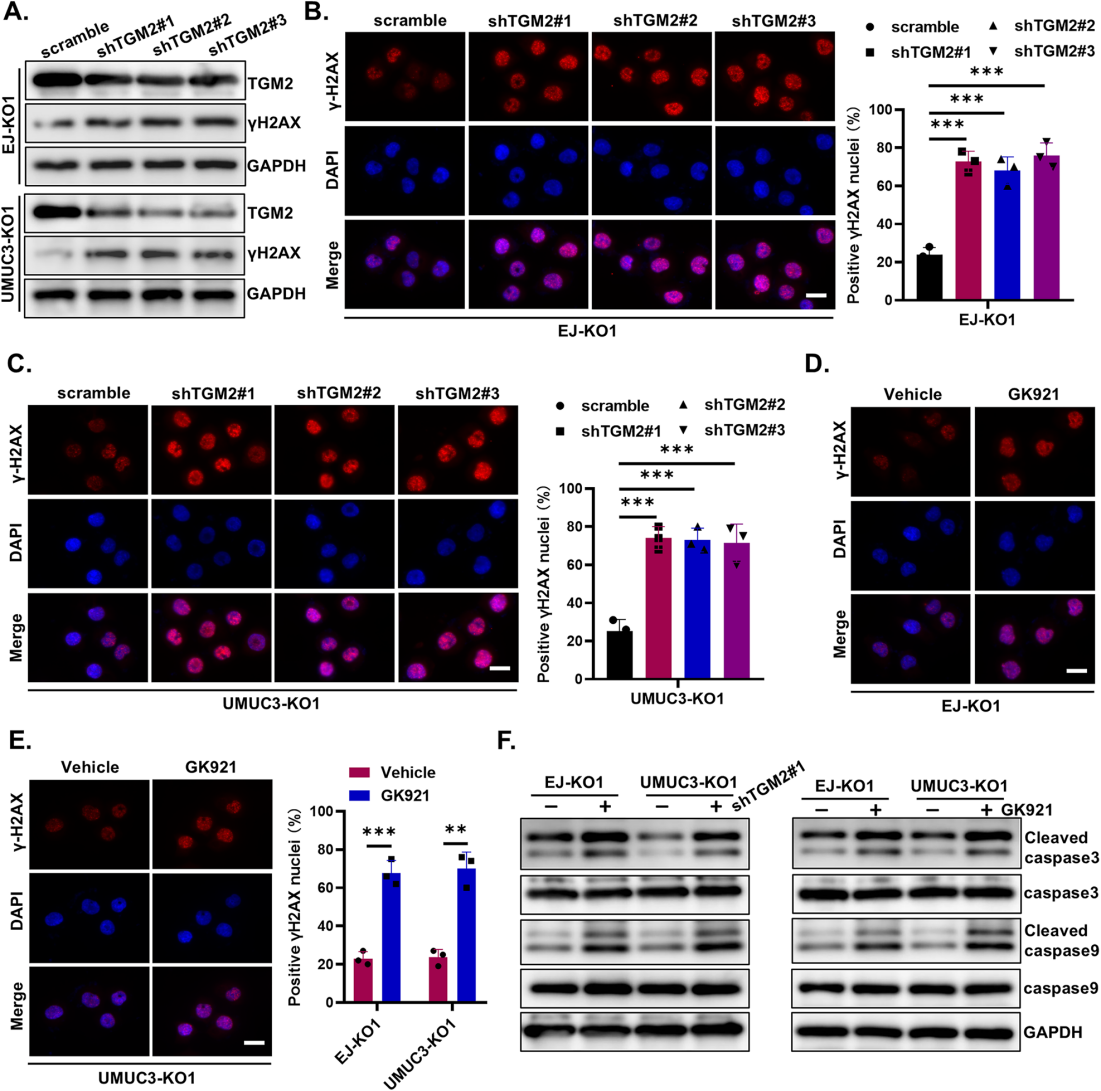
MSH2 loss potentiates chemoresistance via TGM2-mediated enhancement of DNA damage repair. **A** Western blot analysis depicted the expression of γH2AX when MSH2-deficient bladder cancer cells were treated with CDDP (5 μM). Representative immunofluorescence staining showed the expression of γH2AX when MSH2-deficient EJ (**B**) and UMUC3 (**C**) cells stably transfected TGM2 knockdown plasmids were treated with CDDP (5 μM) (*n* = 3), Scale bar indicated 20 μm. Representative immunofluorescence staining showed the expression of γH2AX when MSH2-deficient EJ (**D**) and UMUC3 (**E**) were treated with GK921 (10 μM) and CDDP (5 μM) (*n* = 3), Scale bar indicated 20 μm. **F** Western blot analysis showed the cleaved caspase-3, caspase-3, cleaved caspase-9 and caspase-9 levels in MSH2-deficient cells transfected with TGM2 knockdown plasmid or treated with GK921 (10 μM) upon CDDP (5 μM) treatment. Error bars represent standard deviations of the mean obtained from three independent experiments. **P < 0.01, ***P < 0.001 by *t* test in **E**, one-way ANOVA in (**B**, **C**).

Previous studies have been reported that TGM2 could promote DNA double-strand breaks (DSBs) repair by interacting with DNA repair-related proteins [31, 32]. Therefore, we investigated the mechanism by which TGM2, which was upregulated in MSH2-knockout cells, promoted CDDP resistance. We examined γH2AX, a well-established marker of DNA DSBs. As shown in Fig. 6A–C, TGM2 knockdown increased the expression of γH2AX under CDDP treatment in MSH2-deficient bladder cancer cells. Moreover, GK921, a TGM2-specific inhibitor, could also increase the expression of γH2AX (Fig. 6D, E). The prolonged presence of unrepaired DNA damage may lead to more pronounced downstream effects, including enhanced levels of apoptosis execution proteins [33, 34]. We found that knockdown of TGM2 or GK921 increased the levels of cleaved caspase-9 and cleaved caspase-3 in MSH2-deficient bladder cancer cells treated with CDDP (Fig. 6F). The elevated levels of γH2AX and associated downstream apoptotic activation indicated that TGM2 inhibition exacerbated CDDP-induced DNA DSBs, thereby enhancing the sensitivity of MSH2-deficient bladder cancer cells to CDDP.

## Discussion

Although CDDP-based chemotherapy is the first-line therapy option for advanced and metastatic BCa, the majority of patients eventually become unresponsive after several chemotherapy cycles and suffer tumor relapse. CRISPR-cas9 screens have revealed that low MSH2 expression acts as a potential biomarker to predict resistance to CDDP-based chemotherapy in advanced BCa [9]. Consistently, our previous study has demonstrated that BCa with high MSH2 expression exhibits greater sensitivity to CDDP [10]. However, the specific mechanism by which MSH2 deficiency drives chemoresistance to CDDP in BCa remains poorly understood. In this study, we uncover that the DNA repair protein MSH2 exerts a novel epigenomic function in BCa by altering chromatin architecture. Specifically, knockout of MSH2 induces the loss of TADs around the TGM2 locus, leading to increased chromatin accessibility and enhanced recruitment of the transcription factor AP-1 to the TGM2 promoter. Notably, pharmacological inhibition of TGM2 using GK921, in combination with CDDP, significantly improves therapeutic efficacy, indicating that GK921 may serve as a potential and effective therapeutic agent for MSH2-deficient BCa. More meaningfully, because the gene function of MSH2 in adjacent non-tumor tissues is normal, this approach might increase the therapeutic window of chemotherapeutics, thereby reducing the side effects of chemotherapy drugs.

In recent years, the biological mechanisms underlying cisplatin resistance have been extensively investigated. Among them, the most recognized mechanisms of cisplatin resistance involve enhancement of DNA repair pathways, inhibition of apoptotic cell death, alterations in ABC transporter and maintenance of cancer stemness [35]. Our previous study has demonstrated that circSTX6 promotes the expression of the SUZ12 protein through a dual regulatory mechanism, thereby enhancing DNA damage repair capacity and mediating CDDP chemotherapy resistance in BCa [36]. Moreover, the WTAP/hsa_circ_0008399 complex facilitates TNFAIP3 expression by mediating the m^6^A methylation of its mRNA, leading to the downregulation of caspase-8 and subsequently inducing CDDP resistance in BCa [37]. In ovarian cancer, osteopontin triggers a signaling cascade by engaging the CD44 receptor and activating the PI3K/AKT pathway, which ultimately enhances ABC transporter-mediated drug efflux and promotes CDDP resistance [38]. Additionally, the mitochondrial protein TACO1 elevates mitochondrial reactive oxygen species (mtROS) through upregulation of oxidative phosphorylation (OXPHOS), thereby driving cancer stemness and CDDP resistance [39]. Collectively, these findings collectively illuminate the complex landscape of chemotherapy resistance mechanisms. In the present study, we demonstrate that TGM2 is essential for the survival of bladder tumors lacking MSH2 in the presence of CDDP. Knockdown of TGM2 or GK921 treatment could increase the chemosensitivity in multiple MSH2-deficient BCa models. Mechanistically, TGM2 knockdown or GK921 increases the expression of γH2AX in MSH2-deficient BCa cells upon CDDP treatment. Furthermore, we confirm that TGM2 knockdown or GK921 treatment leads to the activation of caspase-9 and caspase-3 in MSH2-deficient BCa cells following CDDP exposure. Taken together, these results reveal that TGM2 inhibition promotes chemosensitivity in MSH2-deficient BCa by impairing DNA repair capacity.

DNA is a primary target for chemotherapy, and DSBs represent a common form of DNA damage induced by CDDP. Deficiencies in DNA damage repair pathways can lead to the accumulation of genetic mutations, ultimately resulting in cellular senescence or cell death [33]. However, loss of MSH2, a key component of DNA mismatch repair, has been shown to confer resistance to CDDP-based chemotherapy. In our study, we confirm that elevated TGM2-induced DNA damage repair potentiates the chemoresistance of MSH2-deficient bladder cancer. Previous study has shown that TGM2 promotes radiotherapy resistance in cervical cancer by positively regulating its interacting partner POGZ, which facilitates BRCA1 accumulation at DSBs sites to enhance DNA damage repair [40]. In addition, the interaction between TGM2 and TOPOIIα plays a critical role in DNA damage repair and contributes to the resistance of lung cancer to radiotherapy [32]. Cells employ distinct repair mechanisms to address DNA damage. For instance, non-homologous end joining (NHEJ) and homologous recombination (HR) are primarily responsible for the repair of DSBs [41]. However, whether TGM2-mediated DNA damage repair in MSH2-deficient bladder cancer involves in NHEJ or HR machinery still needs to be determined.

TGM2, a transglutaminase enzyme involved in protein cross-linking and cell adhesion to fibronectin, is overexpressed in multiple tumor types and associated with poor survival [42]. The transcriptional regulation of genes is governed by interactions between trans-acting factors (TAFs) and cis-regulatory elements (CREs) [43]. Active CREs are generally located in accessible chromatin regions that can be accessed by nucleases through unwrapping and evicting nucleosomes [44]. Therefore, identification of accessible chromatin regions helps decipher CREs in the genome, which is important for understanding the complicated transcriptional regulatory networks underlying gene expression. The state trajectories of chromatin can be assessed by several established methods, including DNase-Seq, ATAC-Seq and Hi-C [45, 46]. Conventionally, transcriptional regulation of TGM2 has been shown to be associated with changes in the expression levels of certain transcription factors, such as HIF-1α [47], Myc [48], AFF1 [49] and ETS1[50]. Herein, we perform Hi-C analysis and report the first evidence that loss of MSH2 alters 3D genomic features around chr20:36.0Mbp-38.0Mbp and increases chromatin accessibility at TGM2 promoter region, thereby promoting its transcription by recruiting the transcription factor AP-1. MSH2 deficiency-induced upregulation of TGM2 expression could be abrogated by c-Fos/c-Jun knockdown. Moreover, treatment with T-5224, a specific inhibitor of AP-1, effectively abrogates the chemoresistance induced by MSH2 deficiency. However, the mechanism by which knockout of MSH2 increases the spatiotemporal recruitment of AP-1 on TGM2 promoter remains to be further investigated.

In summary, our findings provide comprehensive evidence that MSH2-deficient bladder cancer upregulates chromatin accessibility at the TGM2 promoter, thereby recruiting more transcription factor AP-1 to upregulate TGM2 expression, ultimately promoting DNA damage repair and cisplatin resistance. Notably, inhibition of TGM2 by shRNA technology or treatment with GK921 could augment cisplatin chemosensitivity in MSH2-deficient bladder cancer. Collectively, this study elucidates a novel and promising therapeutic approach for bladder cancer characterized by MSH2 deficiency.

## Materials and methods

### Cell culture and treatment

Human bladder cancer cell line UMUC-3 was purchased from American Type Culture Collection (ATCC, USA). EJ cells were obtained from the Institute of Biochemistry and Cell Biology of Chinese Academy of Sciences (Shanghai, China). UMUC-3 cells were cultured in DMEM (Gibco, USA) medium plus 10% FBS (Gibco, Australia origin), 1% penicillin/streptomycin (Gibco, USA), and EJ cells were cultured in RPMI-1640 (Gibco, USA) medium supplemented with 10% FBS (Gibco, Australia origin) and 1% penicillin/streptomycin (Gibco, USA). All cells were grown in a humidified atmosphere at 37 °C with 5% CO_2_. All cell lines were routinely authenticated and confirmed negative for *Mycoplasma* contamination. Cisplatin (Sigma, USA) was solubilized in DMSO. GK921 (MCE, USA), a TGM2 inhibitor, was solubilized in DMSO. T-5224 (MCE, USA), an AP-1 inhibitor, was solubilized in DMSO. BBN was purchased from TCI (Oregon, USA).

### CRISPR/Cas9 KO

EJ and UMUC3 cells were transfected with the pSpCas9(BB)-2A-Puro (PX459) plasmid (Addgene, USA) containing MSH2 single-guide RNAs (sgRNAs) using Lipofectamine 2000 (Invitrogen, USA) following the manufacturer’s instructions. Oligonucleotides encoding sgRNAs specific for Msh2 were synthesized and inserted into a Lenti-sgRNA-Cas9 single-vector lentivirus (GeneChem, China). The sgRNAs were listed in Supplementary Table S1. MB49 cells were seeded into a six-well plate at a density of 20–30%, and lentiviral particles were added according to the manufacturer’s instructions (MOI = 20). The bladder cancer cells were screened with Puromycin (Invitrogen, USA) for 2 weeks, and then were seeded into 96-well plate. Single-cell colonies were selected, and knockout efficacy was tested by western blot.

### Western blotting

Cellular proteins were extracted with RIPA lysis buffer (Thermo Scientific, USA) according to the instructions. Western blot assay was performed as previously described [37]. Briefly, the protein samples were subjected to SDS-PAGE and subsequently were transferred to PVDF membranes. Then, blocking with 5% non-fat milk, membranes were efficiently incubated with primary and HRP-conjugated secondary antibodies before visualizing bands via Bio Spectrum 600 Imaging System (UVP, USA). Antibodies used included primary antibodies against MSH2 (Abcam, ab70270), TGM2 (Abcam, ab137378), c-Jun (Abcam, ab31419), c-Fos (CST, #2250), γH2AX (CST, #9718), caspase3 (proteintech, 19677-1-AP), caspase9 (proteintech, 10380-1-AP) and GAPDH (proteintech, 10494-1-AP).

### IC50 determination

The MSH2-WT and MSH2-KO cells were seeded into a 96-well plate at a density of 5000 cells per well, and were cultured at 37˚C overnight. Then, the cells were treated with a series of dilute concentrations of cisplatin (0, 1, 2, 4, 6, 8, 10, 12, 14, 16, 18 and 20 µM, Sigma, USA) for 24 h. Afterward, cell viability was measured by the Cell Counting Kit-8 (CCK-8) method (Dojindo, Japan) according to the manufacturer’s instructions. IC50 was calculated by the Probit regression model.

### RT-PCR and quantitative real-time polymerase chain reaction (qRT-PCR)

Total RNA was isolated from cells with FastPure Cell/Tissue Total RNA Isolation Kit V2 (Vazyme, China) according to the manufacturer’s instructions. cDNA was synthesized using HiScript III RT SuperMix (Vazyme, China). The qRT-PCR analyses were performed using SYBR Green Master Mix (Vazyme, China) and primers (Supplementary Table S1). The results of mRNA levels were analyzed by the 2^-△△Ct^ method.

### Apoptosis assay

The cell apoptosis assay was performed according to the manual of FITC Annexin V Apoptosis Detection Kit I (BD, USA). Briefly, cells were seeded into a six-well plate administered with DMSO or CDDP for 24 h. They were harvested and resuspended in 200 μl of binding buffer. Then, 5 μl of Annexin V-FITC and 5 μl of PI were added to the suspensions, and the cells were incubated in the dark at 4 °C for 30 min. Subsequently, the samples were analyzed by Flow Cytometry (Becton Dickinson, USA). PI negative and FITC Annexin V positive represents apoptosis at an earlier stage, and cells that are in late apoptosis or already dead are both FITC Annexin V and PI positive. The apoptosis assay for MB49 cells was determined according to the manual of Annexin V-PE/7-AAD Apoptosis Detection Kit (Vazyme, China). All data were analyzed by FlowJo software (FlowJo).

### Migration assay

For transwell migration assays, a 24-well transwell chamber (Costar, USA) was employed to evaluate cell migration ability. Cells were resuspended in 200 μL of serum-free medium and seeded into the upper chambers at a density of 5 × 10⁴ cells per well. The lower chambers were filled with 600 μL of medium containing 10% FBS as a chemoattractant. Following incubation for 24 h, non-migrated cells on the upper surface of the membrane were gently removed using cotton swabs. Cells that had migrated to the lower surface were fixed with 4% paraformaldehyde and stained with 0.1% crystal violet in PBS. Migrated cells were visualized and photographed under a microscope, and quantified by counting three randomly selected fields per well.

### RNA sequencing

Total RNA was isolated from MSH2-knockout EJ cells with or without CDDP treatment and the corresponding wild-type cells using FastPure Cell/Tissue Total RNA Isolation Kit V2 (Vazyme, China). Transcriptome sequencing on an Illumina HiSeq X Ten platform was carried out by SeqHealth Tech (Wuhan, China). Sequencing results were deposited in the GeneExpression Omnibus database (https://www.ncbi.nlm.nih.gov/geo/query/acc.cgi?acc=GSE193754, https://www.ncbi.nlm.nih.gov/geo/query/acc.cgi?acc=GSE193753).

### Vector construction and cell transfection

Oligonucleotides encoding short hairpin RNAs (shRNAs) specific for TGM2, c-Fos and c-Jun (Supplementary Table S1) were cloned into pLKO.1-puro vector (Sigma, USA). Transfection was carried out using Lipofectamine 2000 (Life Technologies, USA) according to the manufacturer’s instructions. Stable cell lines were obtained by selection with puromycin. Scramble shRNA was applied as controls (Supplementary Table S1).

### Cell Counting Kit-8 (CCK-8) assay

The cell viability of was tested by CCK-8 kit (Dojindo, Japan) following the manufacturer’s instructions. Briefly, cells were cultured in 96-well plates about 5000 per well for 24 h. Then we added 10 μl CCK-8 solution into each well, and samples were incubated for 2 h at 37 °C. The absorbance at 450 nm was captured using spectrometer (Thermo Fisher Scientific, USA).

### Generation and identification of Msh2-knockout Mice

Msh2 knockout mice on the C57BL/6 J background were generated by a CRISPR/Cas9 system from GemPharmatech Co., Ltd. (Nanjing, China). Exons 2 and 3 of Msh2 gene were selected as the target region and were deleted. The genotype of mice was identified by Quick Genotyping Assay kit for mouse tail (Beyotime, China) according to the manufacturer’s instructions. Primers for the PCR ① were as follows: forward: 5′- AACTATCTCGCCAGCCCGAA-3′ and reverse: 5′- GGAGAGTTTGAATGCCATGTTGAG -3′. Primers for the PCR ② were as follows: forward: 5′-TCAGAGTCTCGCTGTGTACTGCCT-3′ and reverse: 5′ - AAACCTGTAAATGCTCAGGGTTCCC-3′.

### BBN-induced autochthonous tumor model

Msh2-knockout mice (Msh2 ^-/-^) (males, 3–4 weeks old) were housed in pathogen-free facilities maintained at 25 °C with a 50–60% relative humidity and a 12-h light/dark cycle. All mice had *ad libitum* access to standard rodent chow and were filtered with water. Each mouse was supplied *ad libitum* with ddH_2_O containing 0.05% BBN (TCI, USA) in opaque bottles for 23 weeks. BBN water was freshly prepared twice a week, and consumption was recorded to assess BBN intake. Mice were randomly allocated into three groups (*n* = 5), (a) Msh2^-/-^ + DMSO; (b) Msh2^-/-^ + CDDP; (c) Msh2^-/-^ + CDDP + GK921; (CDDP, 2 mg/kg, biw; GK921, 2 mg/kg, daily). Orthotopic bladder tumors were monitored by ultrasound imaging at the end of the experiment. All procedures involving the mice were approved by the Animal Care Committee of Tongji Medical College (approval number: 20202586).

### Xenografts in nude mice

BALB/c nude mice (females, 4 weeks old) were purchased from the Beijing Vital River Lab Animal Technology Co., Ltd. Six mice were randomly included in each group, and bladder cancer cells were injected subcutaneously into the left side of the dorsum (3 × 10^6^ cells per mouse). One week after injection, DMSO or CDDP was administered by intraperitoneal injection twice a week at the dose of 2 mg/kg. Meanwhile, GK921 was orally administered daily at the dose of 2 mg/kg. The volumes of tumors were measured with caliper every 3 to 4 days following formula: length × width^2^× 0.5. A month later, the mice were sacrificed and were examined for tumor weight. All animal experiments were carried out in accordance with NIH guidelines for the care and use of laboratory Animals and were approved by the Animal Care Committee of Tongji Medical College (approval number: 20202586).

### Orthotopic bladder tumor model

For orthotopic bladder tumor model, the procedures were performed as described previously [51]. In short, five BALB/c mice (females, 4 weeks old) were randomly included in each group, and were anesthetized with 10% chloral hydrate, and were placed in a supine position on a constant temperature blanket. Then, 24 G intravenous catheter was inserted into the urethra slowly. Subsequently, we injected silver nitrate into the bladder through the catheter and kept it in bladder for 10 s. The bladder was flushed by injecting sterile water. Then, a sterile needle was used to inject the prepared 2 × 10^6^ cells. For drug studies in vivo, DMSO and CDDP were administered by intraperitoneal injection (2 mg/kg for CDDP, biw), and GK921 was supplied at the dose of 2 mg/kg daily. Tumors were monitored by ultrasound imaging at the end of the experiment. Animal experiments were performed in accordance with NIH guidelines for the care and use of laboratory Animals and were approved by the Animal Care Committee of Tongji Medical College (approval number: 20202586).

### Actinomycin D assay for mRNA lifetime

For Actinomycin D treatment, cells were seeded in a 6-well plate at 10^5^ cells per well overnight. Then, the cells were treated with 5 μg/ml Actinomycin D (Sigma, USA) and were collected at indicated time points. The total RNA was extracted by FastPure Cell/Tissue Total RNA Isolation Kit V2 (Vazyme, China) and was analyzed by RT-PCR. The turnover rate and half-life of mRNA were estimated according to our previously published paper [10].

### Hi-C analysis

Paired-end reads obtained by sequencing were processed by ChIA-PET2 following the instructions from [52]. About 5 × 10^5^ cells were collected in the 1.5 ml tube with 1 mL culture medium, and formaldehyde solution was added to achieve a final concentration of 1%. Then, the tube was placed in a Rotator for 15 min at RT. After cross-linking, the remaining formaldehyde was sequestered by adding 27.9 μL of 2 M glycine (Sigma, USA) and continued to rotate for 10 min at RT. Then, the restriction enzyme HaeIII was used for digestion. Biotin-labeled bridge linkers were used to ligate DNA fragments. Purify labled DNA fragments with Dynabeads (Invitrogen, USA). Quantify the recovered DNA. The purified DNA is used directly for cluster generation and sequencing analysis using the Illumina novaseq6000 following the manufacturer’s protocols. HiC-Pro [53] was used to obtain *.allValidPairs* files and merge technical replicates. Merged *.allValPairs* files are down-sampled for consistent sequencing depth. Juicer tools (v1.22.01) [54] and hic2cool (v0.8.3) are used to convert *.allValidPairs* files to *.hic* and *.mcool* files. DcHiC [55] was deployed to identified Hi-C compartments at 100-kb resolution. OnTAD [56] is used to define hierarchy TAD in 50-kb resolution. The Hi-C raw data were deposited in the GeneExpression Omnibus database (https://www.ncbi.nlm.nih.gov/geo/query/acc.cgi?acc=GSE262994, accession number, gpenssecfronzgv).

### Chromatin immunoprecipitation

The chromatin immunoprecipitation (ChIP) assay was performed using ChIP Assay Kit (Sigma, USA). Briefly, bladder cancer cells (1 × 10^7^) were washed with PBS and then fixed with 1% formaldehyde for 10 min, and were quenched with 0.125 M glycine for 5 min at 37 °C. Then, samples were lysed in SDS Lysis Buffer, and cell lysate was sonicated by Sonics Vibra-Cell^TM^ (TOUCHBOX, USA) to shear chromatin DNA to a size range of 200–1000 bp. The supernatant was diluted 10 folds in ChIP Dilution Buffer and was precleared with 60 μL agarose beads for 30 min. The supernatant fraction was immunoprecipitated with indicated antibodies (2 μg) against c-Fos, c-Jun, PAX5 or negative IgG antibody overnight at 4 °C. Antibody-chromatin complexes were pulled down with protein A agarose/salmon sperm DNA beads (Sigma, USA) for 1 h at 4 °C. PCR primers for the TGM2 promoter were listed in Supplementary Table S1. The recovered DNA was detected by PCR analysis. The relative amount of immunoprecipitated DNA was evaluated by standardizing the data to negative control.

### Immunofluorescence assay

Immunofluorescence staining was performed to detect γH2AX in BCa cells. Briefly, cells were seeded in confocal dishes and cultured overnight to allow adhesion. The following day, cells were fixed with 4% paraformaldehyde, permeabilized with 0.5% Triton X-100, blocked with 5% BSA and incubated with γH2AX (CST, USA) primary antibody at 4 °C overnight. After washing twice with PBS and then cells were incubated with the Alexa Fluor 594-conjugated secondary antibody (ABclonal, China) for 1 h at 37 °C in the dark, followed sealing with parafilm containing DAPI. Fluorescence images were captured via a confocal laser scanning microscope (Olympus, China).

### Statistical analysis

All the data statistical analyses were performed using GraphPad Prism 8.1.2 software (La Jolla, USA). All experiments were performed independently at least three times, and the number of independent experiments is reported in the figure legend. Statistical analyses included unpaired two-tailed t-tests to compare differences between two groups and one-way ANOVA to compare differences between multiple groups. Log-rank test was used to assess survival difference. Data were presented as mean ± standard deviation (SD). *P* < 0.05 was considered statistically significant. No statistical methods were used to predetermine sample size.

## Supplementary information

This document contains the supplementary figures and legends for this article.

This PDF contains the original Data of western blots for this article.

This file contains the sequences of primers and oligonucleotides used in this study.

## Data Availability

All relevant data are presented in the main manuscript or supplementary files. The RNA-seq and Hi-C raw data in this study are available at GEO under accession number GSE193754, GSE193753 and GSE262994.
