# Supplementary material for: Inhibition of TGM2 enhances cisplatin sensitivity in MSH2-deficient bladder cancer
Source: Cell Death Discov. 2026 May 28;12:318. doi: 10.1038/s41420-026-03182-z (PMC13402307; doi:10.1038/s41420-026-03182-z)
Supplement: Supplementary file 1 — This document contains the supplementary figures and legends for this article. [file 41420_2026_3182_MOESM1_ESM.pdf]

Fig.S1

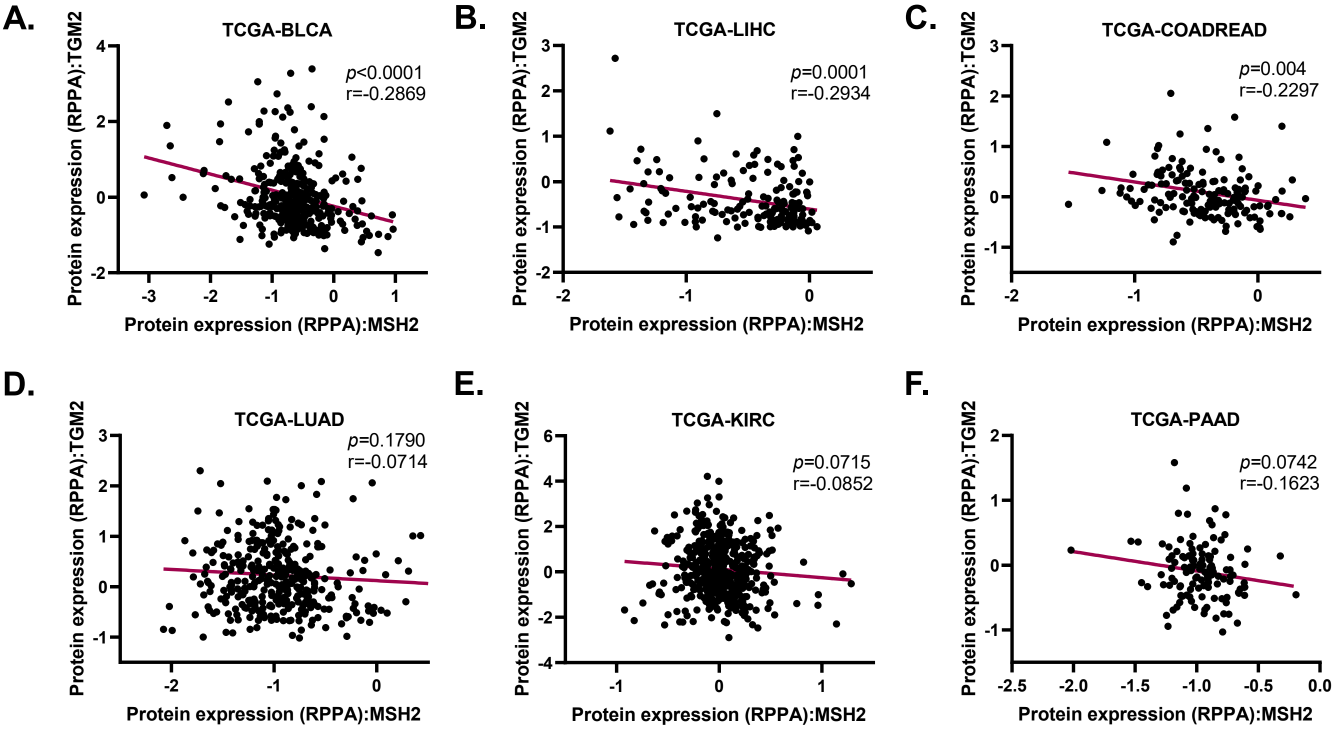

Fig S1. The correlation between TGM2 and MSH2 expression in TCGA cohorts

A Pearson correlation analysis between TGM2 and MSH2 protein expression in TCGA BLCA cohort.

B Pearson correlation analysis between TGM2 and MSH2 protein expression in TCGA LIHC cohort.

C Pearson correlation analysis between TGM2 and MSH2 protein expression in TCGA COADREAD cohort.

D Pearson correlation analysis between TGM2 and MSH2 protein expression in TCGA LUAD cohort.

E Pearson correlation analysis between TGM2 and MSH2 protein expression in TCGA KIRC cohort.

F Pearson correlation analysis between TGM2 and MSH2 protein expression in TCGA PAAD cohort.

Fig.S2

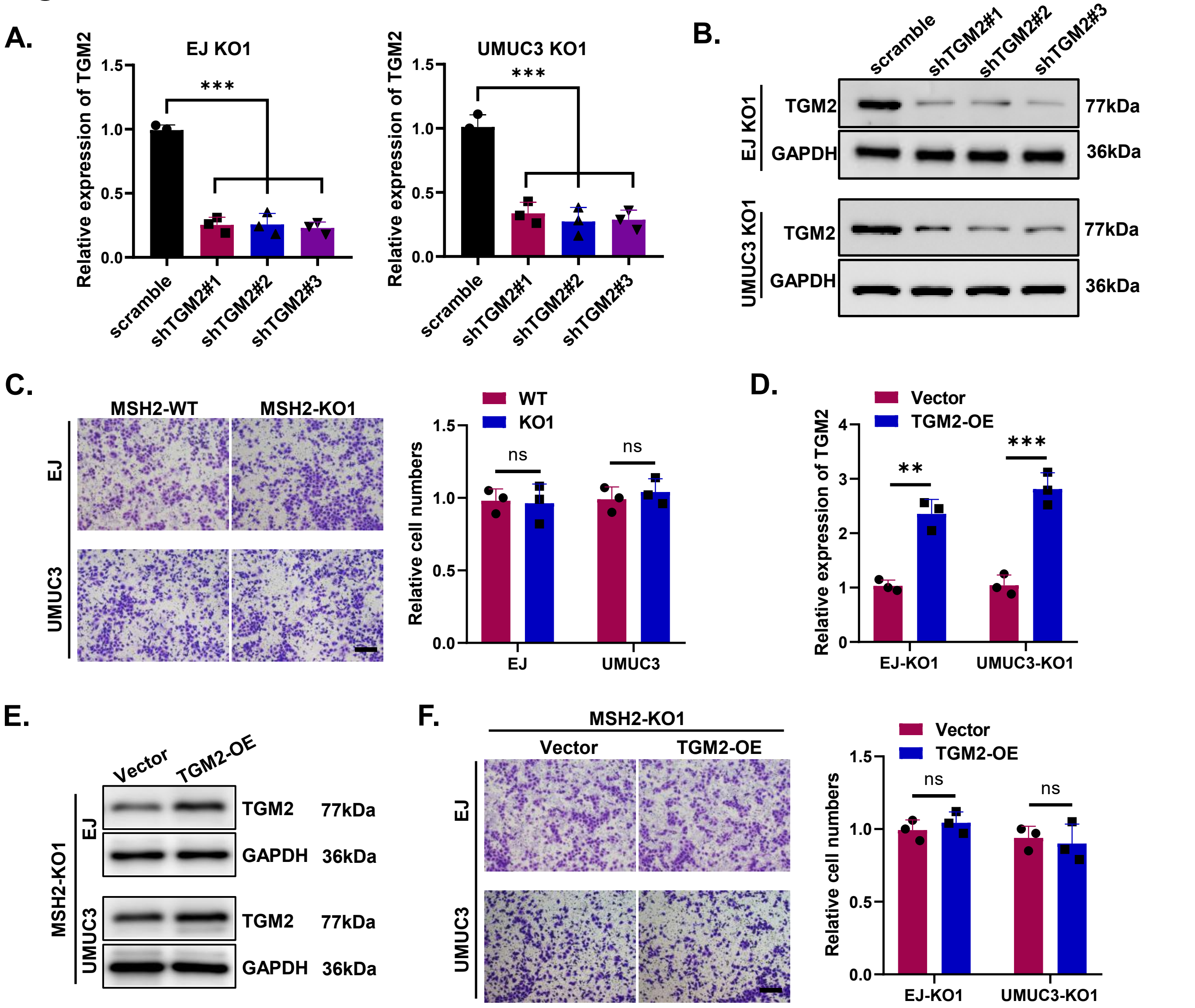

Fig S2. Elevated TGM2 levels did not affect the migration of MSH2-deficient bladder cancer cells

A The knockdown efficacy of TGM2 was detected by qRT-PCR in MSH2-knockout BCa cells (n = 3).

B The knockdown efficacy of TGM2 was detected by western blot in MSH2-knockout BCa cells.

C Representative and quantified results of the Transwell migration assays in the WT and MSH2-KO1 group (n = 3). Scale bar, 100 μm.

D-E The overexpression efficacy of TGM2 was detected by qRT-PCR and western blot in MSH2-knockout BCa cells (n = 3).

F Representative and quantified results of the Transwell migration assays in the Vector and TGM2-OE group in MSH2-knockout BCa cells (n = 3). Scale bar, 100 μm.

Error bars represent standard deviations of the mean obtained from three independent experiments. $^{**}P < 0.01$, $^{***}P < 0.001$ by *t* test in C, D and F, one-way ANOVA in A.

Fig.S3

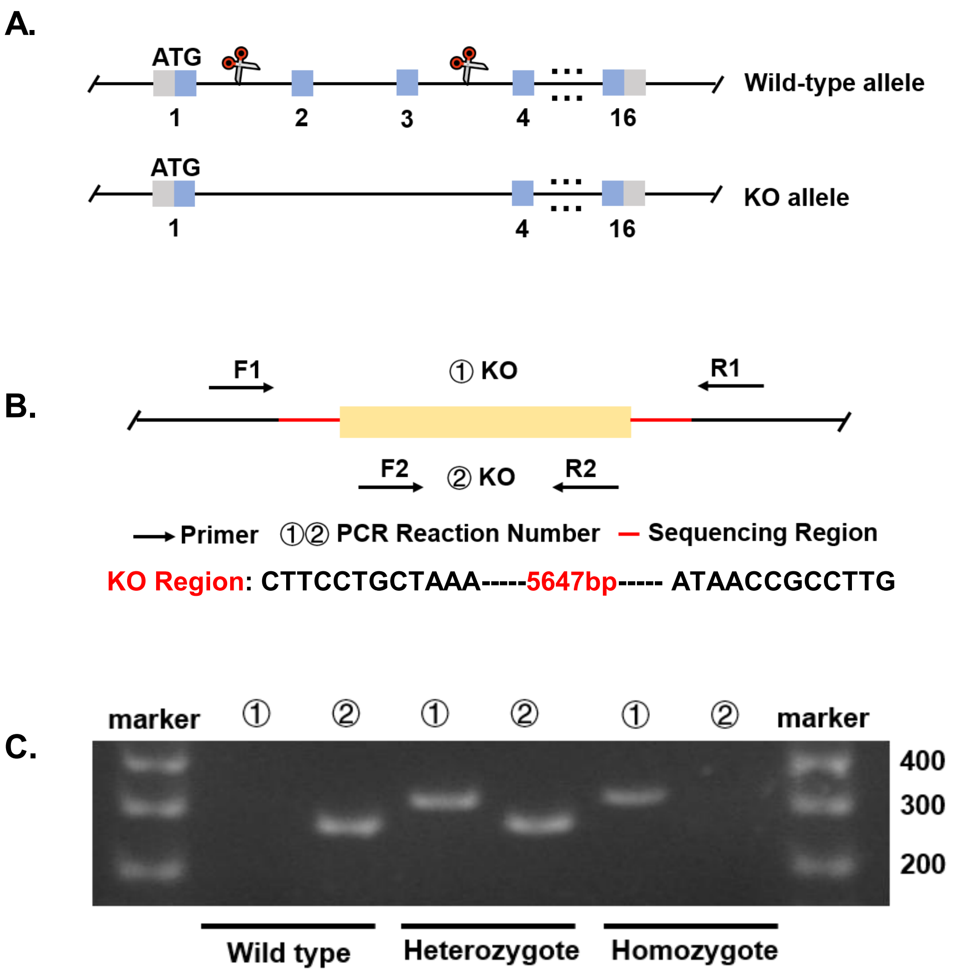

Fig S3. Schematic diagram depicted the identification method of mice genotype

A Schematic diagram showed the knockout site of Msh2 gene.

B-C Wild type: ① PCR reaction without product; ② PCR reaction obtains a single of about 253bp. Heterozygote: ① PCR reaction obtains a single band of about 297bp; ② PCR reaction obtains a single of about 253bp. Homozygote: ① PCR reaction obtains a single band of about 297bp; ② PCR reaction without product.

Fig.S4

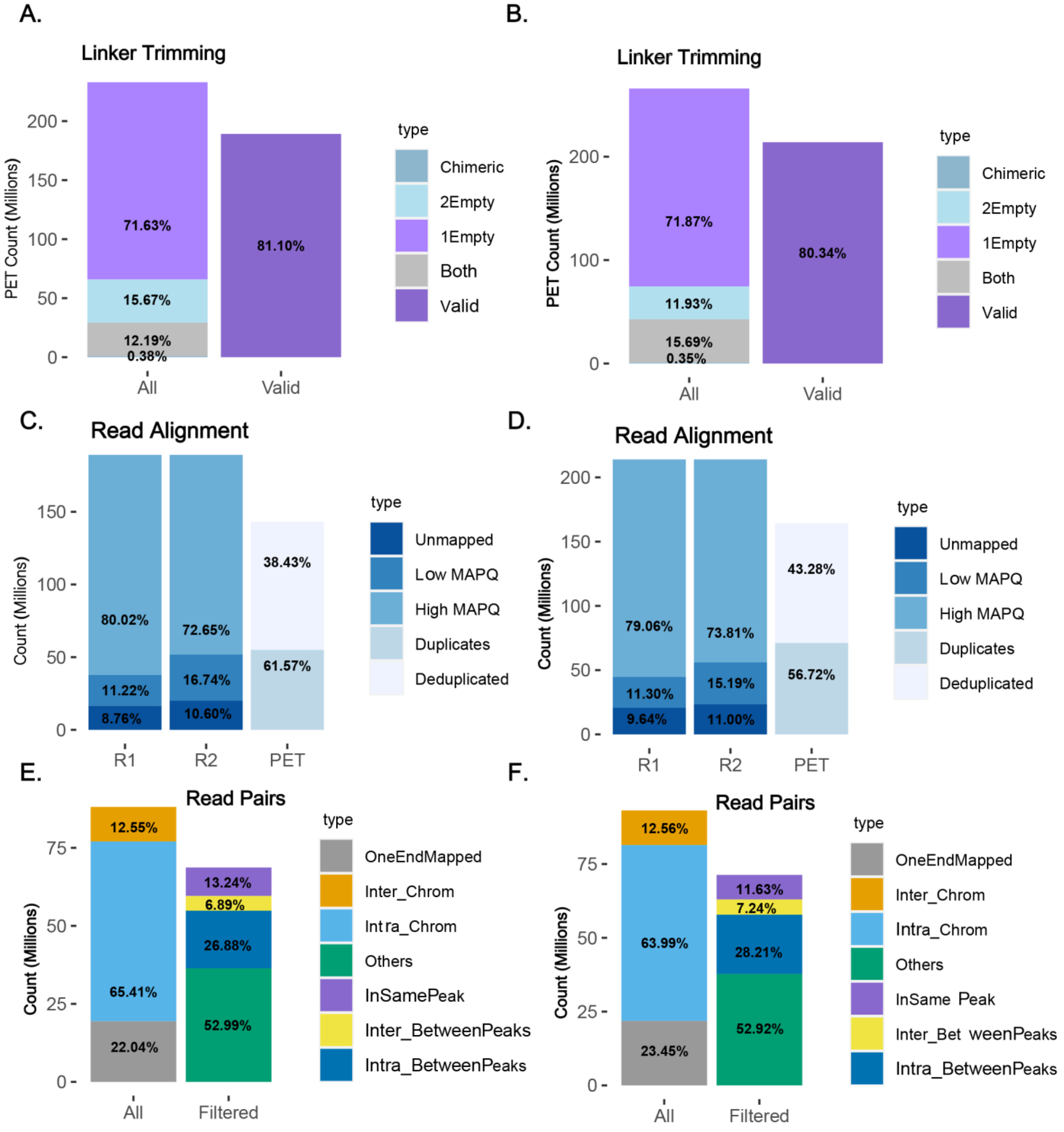

Fig S4. Quality control data for Hi-C sequencing analysis

A Linker Trimming state of BL-HiC sequencing MSH2-WT1 groups.

B Linker Trimming state of BL-HiC sequencing MSH2-KO1 groups.

C Linker mapping quality of BL-HiC sequencing of MSH2-WT1 groups.

D Mapping quality of BL-HiC sequencing of MSH2-KO1 groups.

E Mapping pairs of BL-HiC sequencing of MSH2-WT1 groups.

F Mapping pairs of BL-HiC sequencing of MSH2-KO1 groups.

Fig.S5

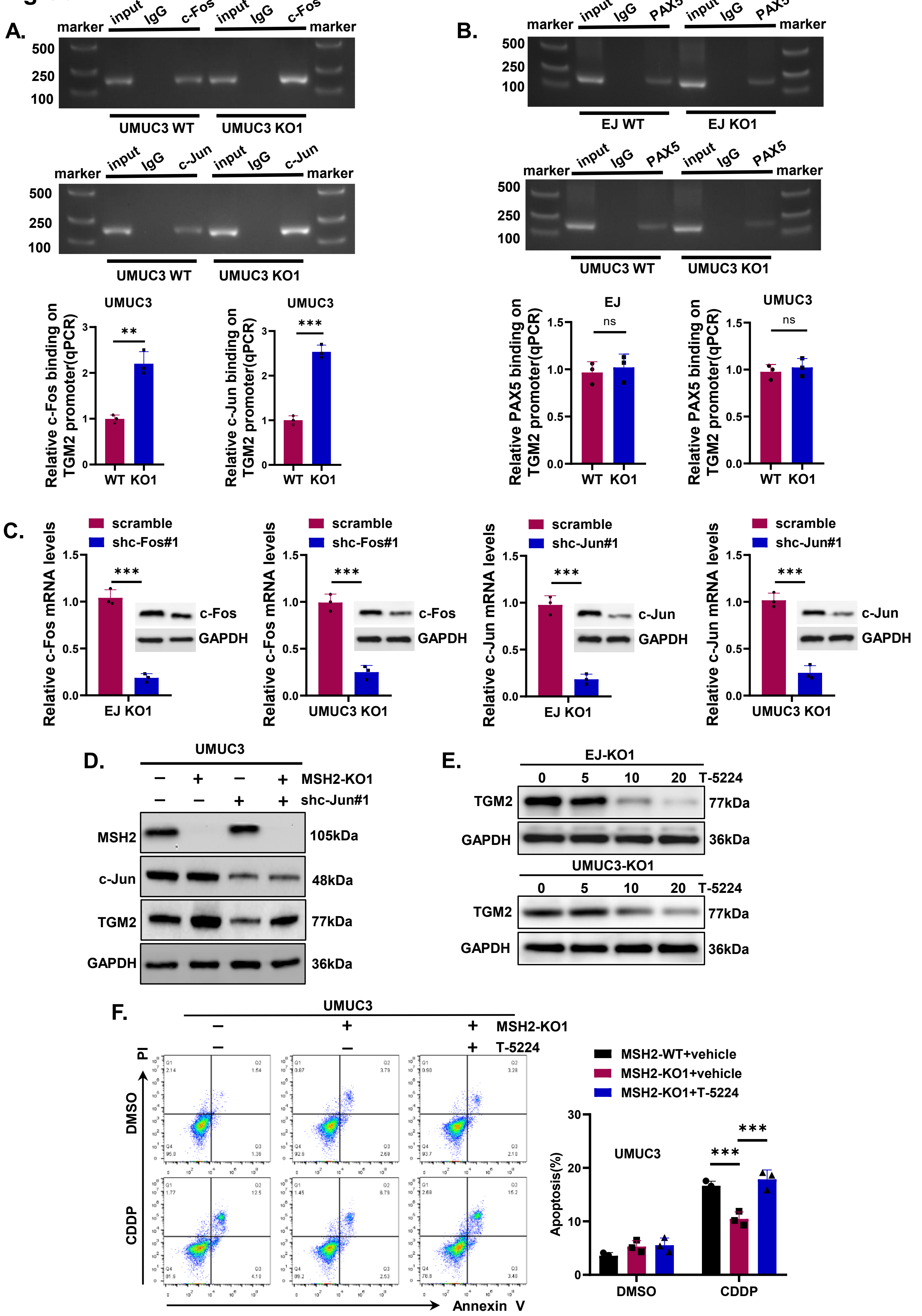
A.
marker input IgG c-Fos input IgG c-Fos marker
500
250
100
UMUC3 WT UMUC3 KO1
marker input IgG c-Jun input IgG c-Jun marker
UMUC3 WT UMUC3 KO1
UMUC3
Relative c-Fos binding on TGM2 promoter(qPCR)
**
WT KO1
Relative c-Jun binding on TGM2 promoter(qPCR)
***
B.
marker input IgG PAX5 input IgG PAX5 marker
EJ WT EJ KO1
UMUC3 WT UMUC3 KO1
EJ
Relative PAX5 binding on TGM2 promoter(qPCR)
ns
C.
scramble
shc-Fos#1
shc-Jun#1
Relative c-Fos mRNA levels
Relative c-Jun mRNA levels
c-Fos
c-Jun
GAPDH
EJ KO1
UMUC3 KO1
D.
UMUC3
MSH2-KO1
shc-Jun#1
MSH2 105kDa
c-Jun 48kDa
TGM2 77kDa
GAPDH 36kDa
E.
EJ-KO1
UMUC3-KO1
0 5 10 20 T-5224
F.
MSH2-KO1
T-5224
PI
DMSO
CDDP
Annexin V
MSH2-WT+vehicle
MSH2-KO1+vehicle
MSH2-KO1+T-5224
Apoptosis(%)
UMUC3
DMSO CDDP

Fig S5. MSH2 deficiency promotes chemoresistance through AP-1/TGM2 axis

A ChIP and qPCR assays showed the changes in binding of c-Fos to TGM2 promoter in MSH2-WT and MSH2-KO UMUC3 cells, and the changes in binding of c-Jun to TGM2 promoter in MSH2-WT and MSH2-KO UMUC3 cells (n = 3).

B ChIP and qPCR assays showed the changes in binding of PAX5 to TGM2 promoter in MSH2-WT and MSH2-KO EJ and UMUC3 cells (n = 3).

C The knockdown efficacy of AP-1 was detected by qRT-PCR and western blot in MSH2-knockout BCa cells (n = 3).

D Western blot assays showed the TGM2 protein levels in MSH2-WT or MSH2-KO UMUC3 cells, and those transfected with scramble or shc-Jun#1.

E Western blot assays showed the TGM2 protein levels in MSH2-WT and MSH-KO1 bladder cancer cells treated with a series of concentrations of T-5224 (0, 5, 10, 20μM).

F MSH2-WT and MSH-KO1 UMUC3 cells were treated with vehicle or T-5224 at a concentration of 10μM. Annexin-V plus PI staining analysis showed the cell apoptosis rate in UMUC3 cells treated with DMSO or CDDP (5μM) for 24h (n = 3).

Error bars represent standard deviations of the mean obtained from three independent experiments. **P < 0.01, ***P < 0.001 by *t* test in A, B, C and one-way ANOVA in F.
