# Supplementary figures and images for: Inhibition of TGM2 enhances cisplatin sensitivity in MSH2-deficient bladder cancer
Source: Cell Death Discov. 2026 May 28;12:318. doi: 10.1038/s41420-026-03182-z (PMC13402307; doi:10.1038/s41420-026-03182-z)

Fig.1B

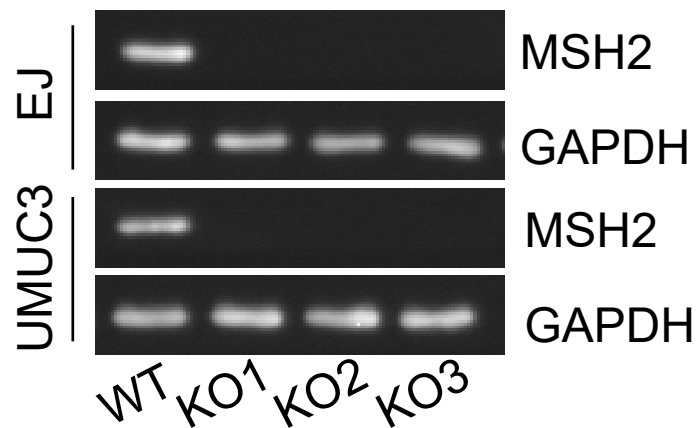

Fig.2D

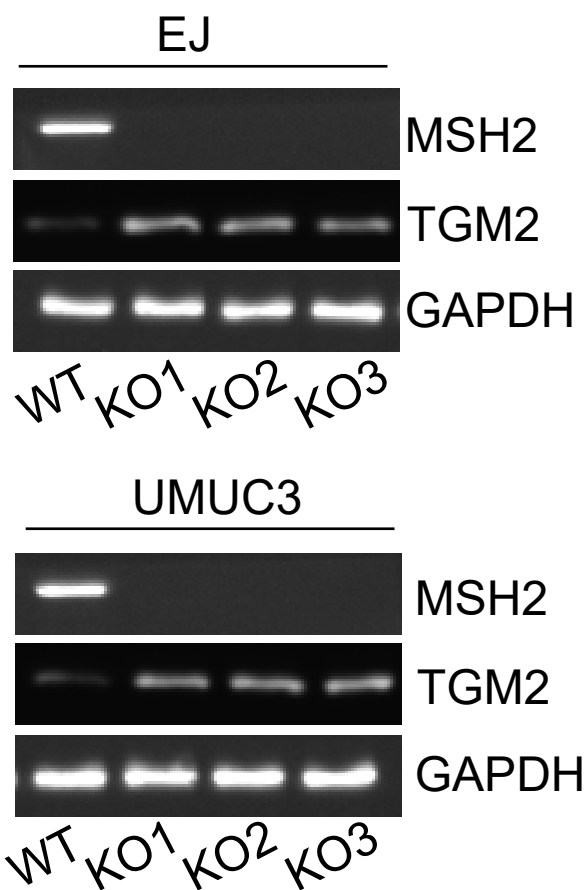

Fig.2F

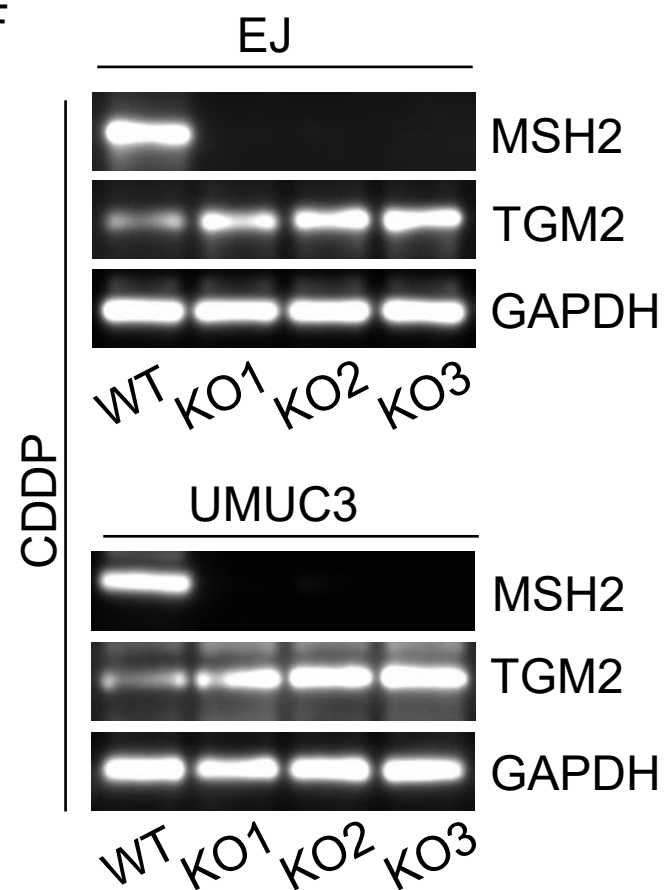

Fig.4A

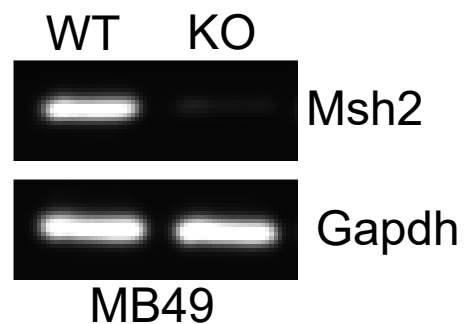

Fig.4D

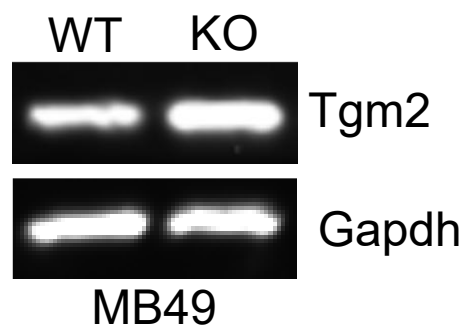

Fig.4E

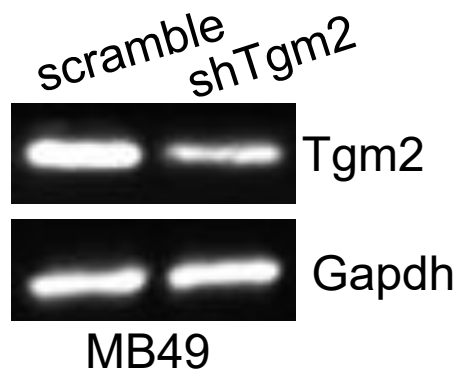

Fig.5F

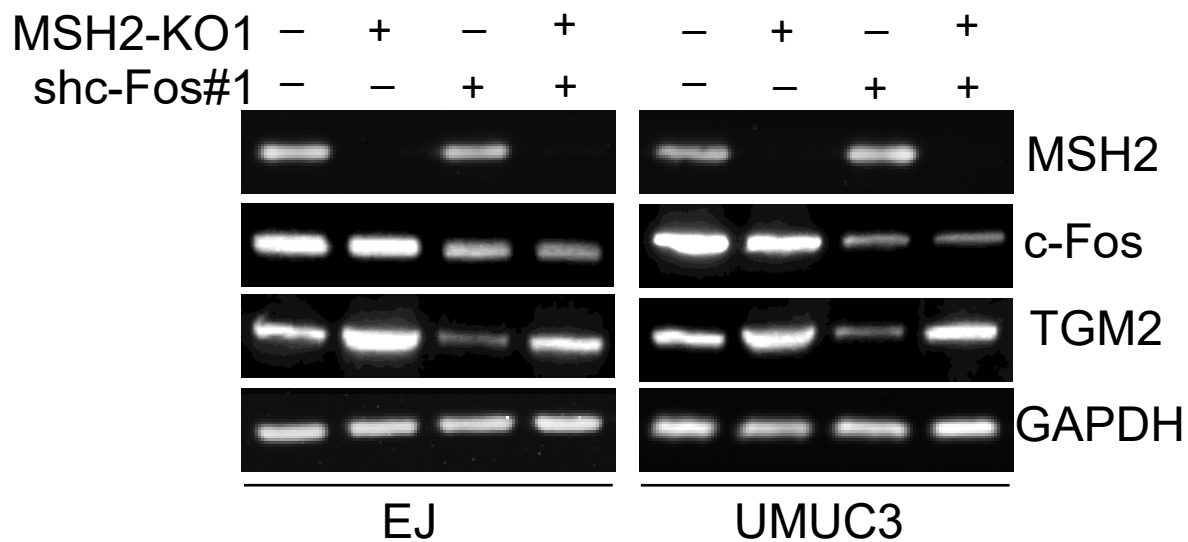

Fig.5G

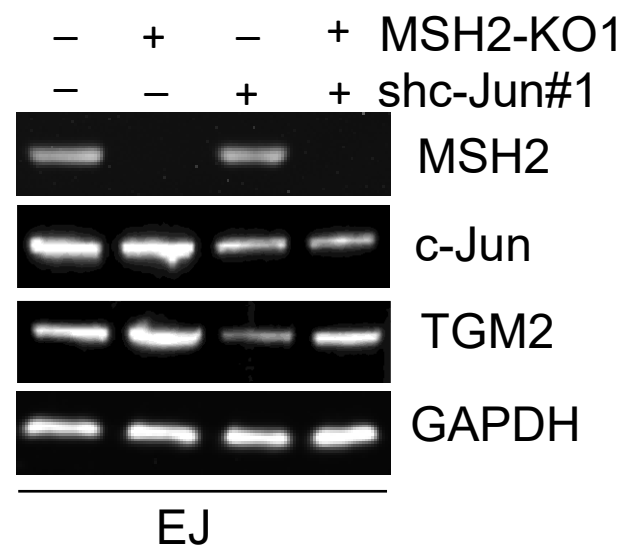

Fig.6A

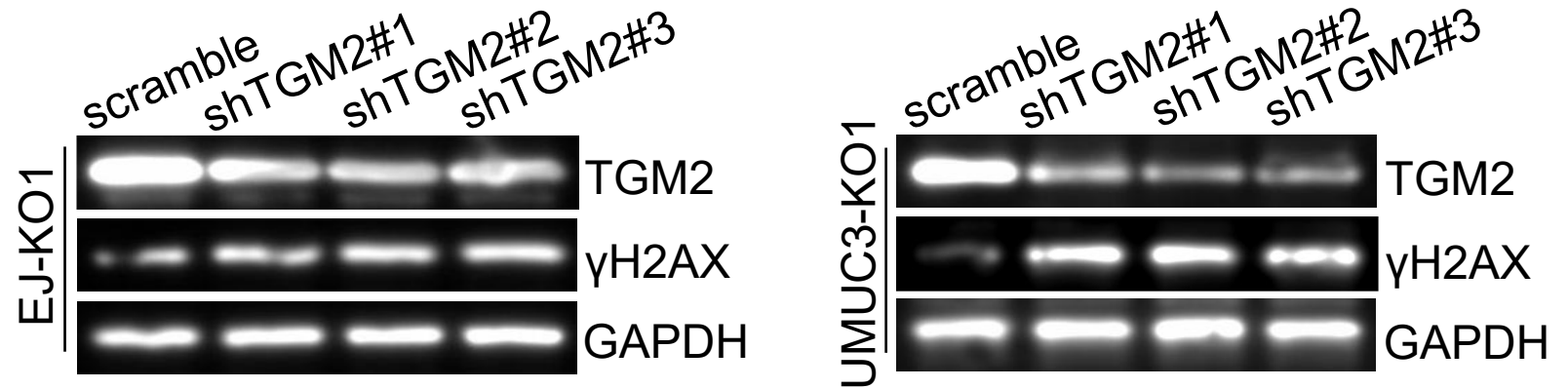

Fig.6F

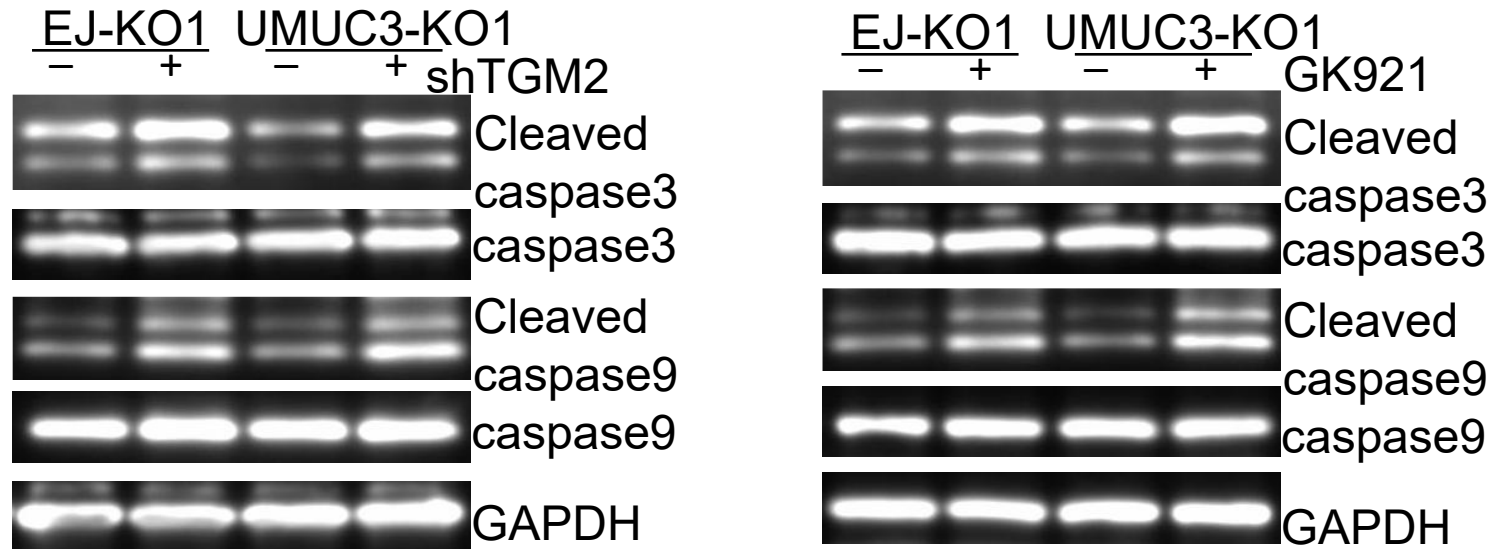

Fig.S2B

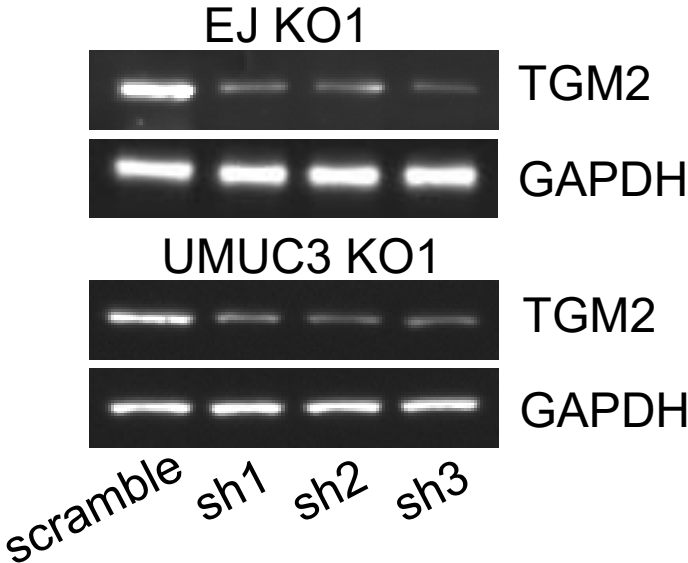

Fig.S2E

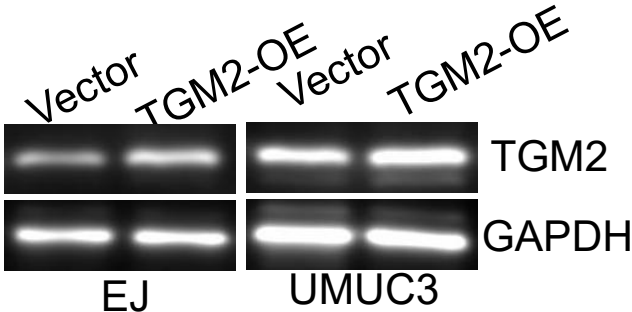

Fig.S5C

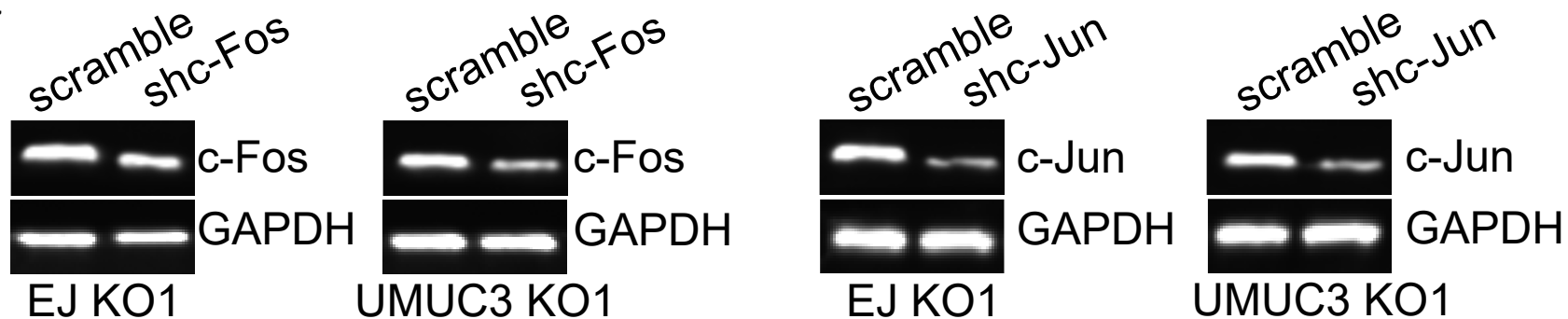

Fig.S5D

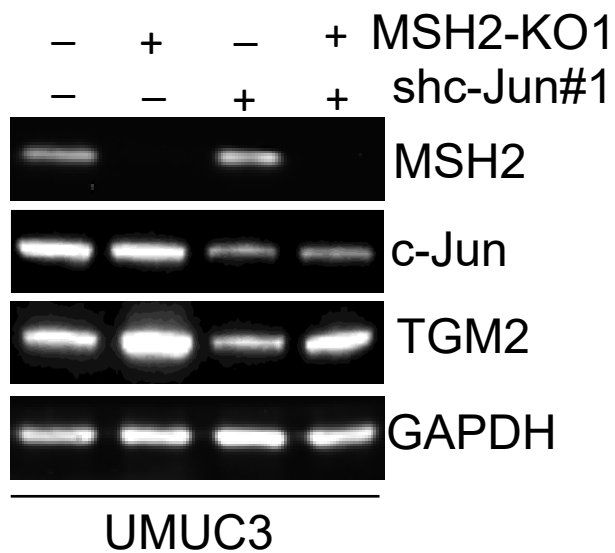

Fig.S5E

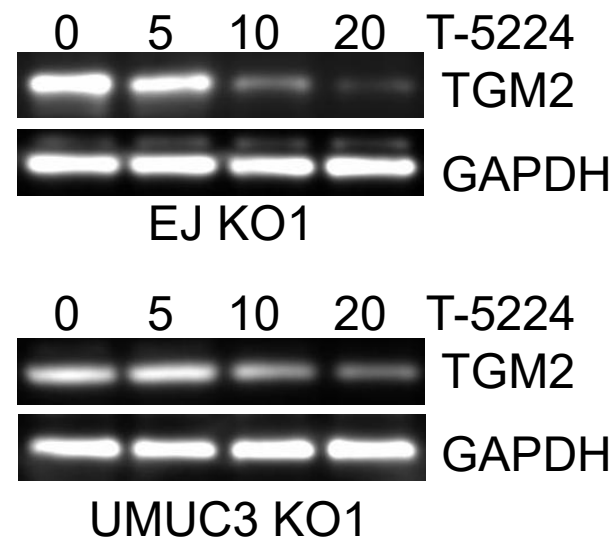

Supplement: Supplementary file 2 — This PDF contains the original Data of western blots for this article. [file 41420_2026_3182_MOESM2_ESM.pdf]
