# Supplementary material for: Inhibition of TGM2 enhances cisplatin sensitivity in MSH2-deficient bladder cancer
Source: Cell Death Discov. 2026 May 28;12:318. doi: 10.1038/s41420-026-03182-z (PMC13402307; doi:10.1038/s41420-026-03182-z)
Supplement: Supplementary file 3 — This file contains the sequences of primers and oligonucleotides used in this study. [file 41420_2026_3182_MOESM3_ESM.docx]

| **Supplementary table 1: The sequences of primers and oligonucleotides used in this study.** | |
| --- | --- |
| **Primers for PCR（5’-3'）** | |
| **TGM2 F** | **TTCTGGCTGACCCTGCACTTT** |
| **TGM2 R** | **ACCCTCCTCCACAGCATCTCTT** |
| **C-Fos F** | **TTACTACCACTCACCCGCAGAC** |
| **C-Fos R** | **TGGGAATGAAGTTGGCACTGGA** |
| **C-Jun F** | **CACGTGAAGTGACGGACTGT** |
| **C-Jun R** | **CCCGTTGCTGGACTGGATTA** |
| **GAPDH F** | **AAATCAAGTGGGGCGATGCTG** |
| **GAPDH R** | **GCAGGAGGCATTGCTGATGAT** |
| **shRNA sequence** |  |
| **scramble** | **TTCTCCGAACGTGTCACGTTTCAAGAGAACGTGACACGTTCGGAGAATTTTTT** |
| **shTGM2#1 or shTgm2#1** | **GTATCACCCACACCTACAAATACTCGAGTATTTGTAGGTGTGGGTGATATTTTT** |
| **shTGM2#2** | **GTTGTGCTGGGCCACTTCATTTCTCGAGAAATGAAGTGGCCCAGCACAATTTTT** |
| **shTGM2#3** | **CCACCCACCATATTGTTTGATCTCGAGATCAAACAATATGGTGGGTGGTTTTT** |
| **shc-Fos#1** | **GCAGATCTGTCCGTCTCTAGTCTCGAGACTAGAGACGGACAGATCTGCTTTT** |
| **shc-Jun#1** | **GGAACAGGTGGCACAGCTTAACTCGAGTTAAGCTGTGCCACCTGTTCCTTTTT** |
| **CHIP TGM2 F** | **AGGCCGAGGGAGGGATGGATG** |
| **CHIP TGM2 R** | **TAATCCTCCGATCTCACCCAT** |
| **MSH2 sgRNA sequence** | |
| **sgRNA1** | **GATCAAGTACATGGGGCCGGC** |
| **sgRNA2** | **GAAGTTTATAAGAATAGAGC** |
| **Msh2 sgRNA#1** | **TGATCAGTTCTCCAATCTCG** |
